# Recent advances in emerging integrated anticorrosion and antifouling nanomaterial-based coating solutions

**DOI:** 10.1007/s11356-024-33825-6

**Published:** 2024-06-04

**Authors:** Paul Thomas, Bichitra Nanda Sahoo, Peter James Thomas, Martin Møller Greve

**Affiliations:** 1 Department of Physics and Technology, University of Bergen, Allégaten 55, 5020 Bergen, Norway; 2 NORCE Norwegian Research Centre AS, Bergen, Norway

**Keywords:** Antifouling, Multifunctional coating, Anticorrosion, Nanocoating

## Abstract

The rapid progress in the marine industry has resulted in notable challenges related to biofouling and surface corrosion on underwater infrastructure. Conventional coating techniques prioritise individual protective properties, such as offering either antifouling or anticorrosion protection. Current progress and innovations in nanomaterials and technologies have presented novel prospects and possibilities in the domain of integrated multifunctional coatings. These coatings can provide simultaneous protection against fouling and corrosion. This review study focuses on the potential applications of various nanomaterials, such as carbon-based nanostructures, nano-metal oxides, polymers, metal–organic frameworks, and nanoclays, in developing integrated multifunctional nano-based coatings. These emerging integrated multifunctional coating technologies recently developed and are currently in the first phases of development. The potential opportunities and challenges of incorporating nanomaterial-based composites into multifunctional coatings and their future prospects are discussed. This review aims to improve the reader’s understanding of the integrated multifunctional nano-material composite coating design and encourage valuable contributions to its development.

## Introduction

The adhering and attaching of living organisms and debris to the submerged surface of water are referred to as biofouling. Biofouling has significant deleterious impacts on both the economy and ecosystem. The prime example is biofouling on ships or vessels, leading to elevated fuel consumption due to the consequent rise in water resistance (Weber and Esmaeili 2023; Kim et al. 2024). Furthermore, the biofouling organism accumulation increases the weight, exacerbating the adverse effects. Inaddition, aquaculture is adversely affected by biofouling, as it hampers the exchange of water and obstructs net meshes, impeding this industry’s overall productivity (Zhang and Qu 2022). Fouling organisms can be transported globally by ships, potentially instigating bioinvasion upon their arrival in a new marine ecosystem devoid of indigenous predators (Lebret et al. 2009). The mobility of organisms to the new ecosystem may cause potential challenges to the species already present in that ecological environment (Vuong et al. 2023). Colonising fouling organisms on surfaces promotes localised alterations in the composition and amounts of oxygen, pH, and ions (Videla 2002). These changes can potentially initiate the degradation of coatings, elevate liquid conductivity, and stimulate chemical and electrochemical processes, which could lead towards corrosion. The corrosion phenomenon that occurs through microorganisms is called biocorrosion or microbially influenced corrosion (MIC). According to estimates, almost 20% of the corrosion observed in aqueous environment can be attributed to microbiologically influenced corrosion (Knisz et al. 2023). Seawater, characterised by a saline level of around 35%, possesses significant corrosive properties that lead to the initiation of chemical and electrochemical processes on subsea surfaces. Marine corrosion has the potential to initiate the formation of little fractures on surfaces, which over time can progress into extensive corrosion, therefore diminishing the structural integrity of the surface and giving rise to significant security concerns (Bhandari et al. 2015; Alcántara et al. 2017). The global maritime industry and naval forces incur substantial financial losses of up to billions of US dollars ascribed to the biofouling phenomenon affecting oceanic facilities, and the global market for corrosion inhibitors is anticipated to be worth US$ 15.51 billion by 2030, up from US$ 10.8 billion in 2022 (Report 2022).

**Fig. 1:**
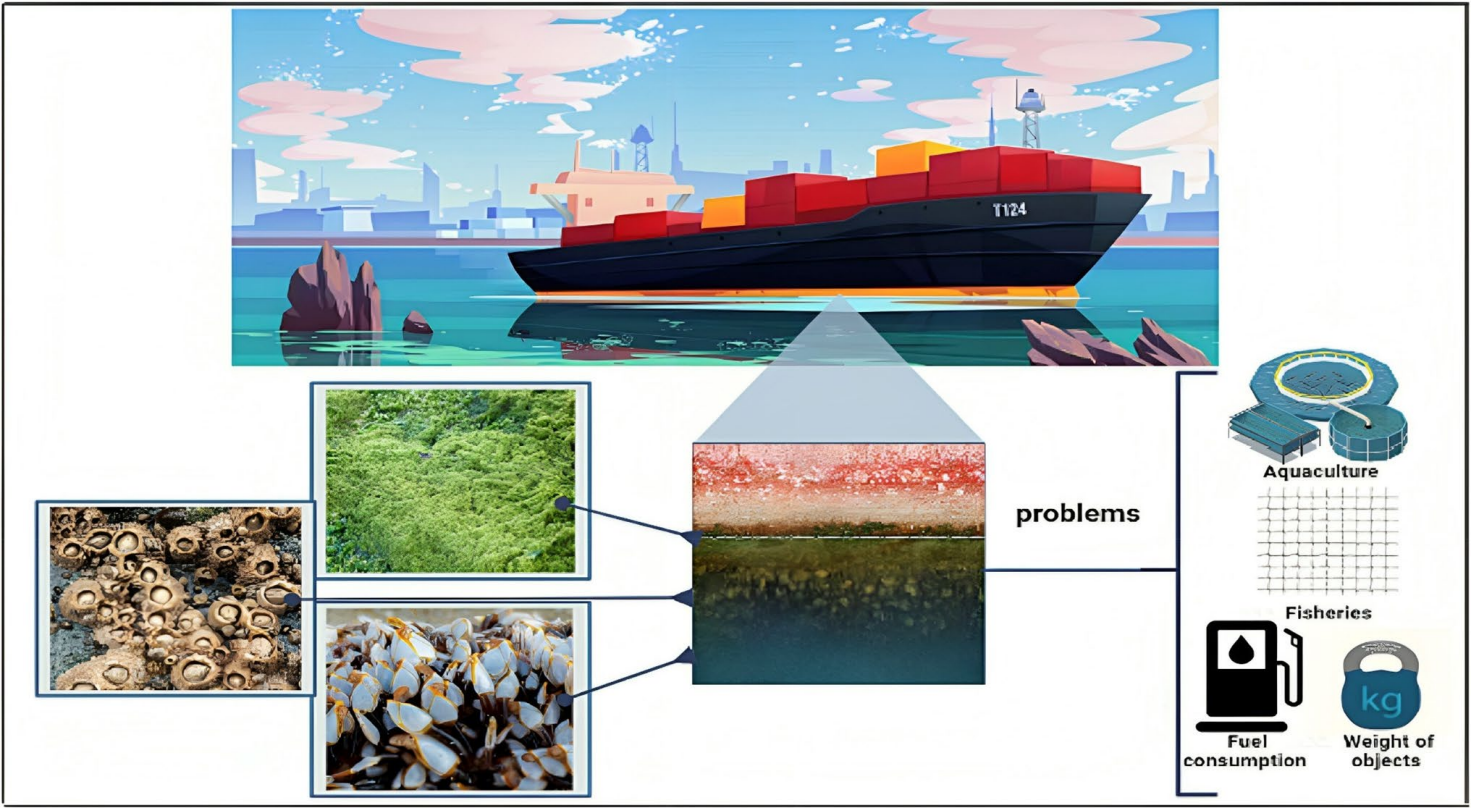
Destructive economic impact of biofouling and corrosion and related problems associated with it (increased fuel consumption, introduction of new species to the ecosystem, and attachments resulting in corrosion and stability) (Karyani et al. 2023)

Industries employ antifouling coatings on exposed surfaces as a preventive measure against biofouling. Recently, chemical antifouling paints and other biocides impeding biofouling have encountered restrictions or outright prohibitions due to their adverse environmental impacts (Korkmaz et al. 2023). Most antifouling coatings often include harmful inorganic substances, such as copper, organic biocides, such as isothiazolone, which are released from the coating and have lethal effects on biofouling organisms (Lagerström et al. 2022; Almeida et al. 2023). These may lead to potential consequences encompass alterations in the composition of communities, diminished species diversity, and disturbances to ecological processes. Although tributyltin self-polishing copolymer paints are often used as antifouling paint, their use is prohibited due to their toxicity. Unfortunately, the market’s non-toxic antifouling options are generally more expensive and less effective than standard biocidal treatments (Silva et al. 2021). Consequently, there is an urgent need for innovative antifouling coatings that are ecologically benign and devoid of detrimental impacts. The development of more environmentally favourable alternatives has thus been given prominence (Fig. 1).

Various methods, including coating, cathodic protection, hot-dip galvanisation, etc., have been commonly employed to prevent corrosion and fouling on the metal (Solovyeva et al. 2023). The hot-dip galvanisation procedure entails immersing steel in a bath of molten zinc. The iron in the steel undergoes a chemical reaction with the zinc, forming a strongly bonded alloy coating that functions as a protective layer. Cathodic protection is an electrochemical approach to prevent corrosion. It is commonly used to preserve metals and marine hulls from corrosion and fouling. Sacrificial anodes are commonly used as cathodic protection for ship hulls; they operate as a protective layer that corrodes before the hull does. In addition to the sacrifical anode, impressed current cathodic protection is also used, and an external power supply is useful to deliver electric current to prevent corrosion. However, coating technique is a commonly employed method amongst all available protection strategies. Numerous coating techniques are available for corrosion and fouling protection however only selective techniques are reliable and efficient (Bijapur et al. 2023). Coating with paints that include mettalic corrosion inhibitors are extensively used in industries as a protective layer for metals to prevent corrosion and fouling. Currently, coatings consisting of metal oxides containing corrosion inhibitors are one of the most promising prevention techniques (Anjum et al. 2020). In addition, various techniques like as thermal spray, sol–gel, physical vapour deposition, microarc oxidation, chemical vapour deposition, electrodeposition, and others developed in laboratory scale show promising for preventing corrosion (Fotovvati et al. 2019). Each of these approaches has its own set of advantages and disadvantages depending on the choice of the material. The wetting of the oxide particle is crucial in certain processing methods used to address the bonding between the particle and the matrix. Moreover, elevated temperatures during specific processing methods can lead to a chemical reaction between the metal matrix and the distributed secondary phase particles. As a result, the stability of the interface and its properties become significantly influential in the protection process. Table 1 lists various techniques employed for assessing the corrosion protection characteristics.

**Table 1:** Various techniques employed for assessing the corrosion protection characteristics

Corrosion test	Potentiodynamic polarization technique	Electrochemical impedance spectroscopy (EIS)	Weight loss	Iron loss measurement
Technique	Based on electrochemistry	Based on electrochemistry	Physical technique	Optical analysis technique
Analysis	Electrochemical cell-based analysis based on working electrode, reference, and counter electrode	Electrochemical cell-based analysis based on working electrode, reference, and counter electrode	Compare the weight of the substrate before and after corrosion	Atomic absorption spectroscopy
	Potentiostat instruments used	Potentiostat instruments used	Estimates corrosion rate in miles per year	Assess the solution of the material submerged
	Polarisation curves are attained by applying the potential of ± 250 mV with respect to the open circuit potential	Parameters that need to be included are initial and final frequency, Points/decade, AC Voltage, and Initial delay		
	Obtains Tafel Plot contains cathodic and anodic branches			
	Anodic branch M → M^2+^ + 2e^−^			
	Cathodic branch 2H^+^ + 2e^−^ → H_2_			
Findings	Tafel Plot-based analysis was used to determine corrosion parameters	Long-term corrosion protection of the material can be assessed	Mpy = 534*W*/*DAT*	Provides iron percentage present in the solution to determine the corrosion occurrence
	Using inbuilt software to identify tafel slopes at anodic and cathodic branches to assess intersection points by drawing tangent lines to estimate E_corr_ and I_corr_	Bode plots (impedance vs frequency on a log scale) and Nyquist plots (actual impedance vs imaginary impedance) were obtained	Mpy- Miles per year	
	E_corr_—Corrosion potential (mV)	We need to fit the Nyquist plot to an equivalent circuit with the help of software to estimate parameters	Corrosion rate (mm·year^−1^, mm a^−1^, Mpy)	
	I_corr_—Corrosion Current density (A.cm^−2^, μA/cm^2^)	R_s_ -Solution resistance	W- Weight loss	
	D-density of specimen	R_f_—Coating resistance (Ω.cm^2^)	A- Area of specimen	
	A- Area of specimen	R_*ct*_—Charge transfer resistance (Ω.cm^2^)	D-density of specimen	
	T- Exposure time		T- Exposure time	
	It- Corrosion current (mA.cm^−2^, μA/cm^2^)	Z- Occurrence of active corrosion calculated from equivalent circuit components (from impedance) (Ω)		
	Rp- polarization resistance (Ω.cm^2^) from i_corr_ estimate the corrosion rate (CR) in different units, as below:			
	CR (in mils per year—mpy) = 0.13 × i_corr_ x [equivalent weight / density]			

Nanotechnology-based surface coating methods have exhibited potential as cost-effective and efficient approaches for safeguarding subsea surfaces from corrosion and biofouling (Gizer et al. 2023; Patil et al. 2023). The dimensions or sizes of nanomaterials, along with their densely layered structure, contribute to adequate bonding and optimal physical coverage of the coated surface. The use of hybrid nanocomposites, including organic–inorganic elements, is an exciting possibility for integrating the distinctive properties of diverse materials, hence offering potential strategies for mitigating biofouling and preventing corrosion (Saravanan et al. 2016). Although nano coatings may appear expensive initially during their production and processing stages, they are typically less expensive over time, mainly when used on a large scale. This is because of the significant savings from maintenance costs, safety, protection against equipment damage, natural resources, and other factors. Moreover, nanostructured metal oxides, such as ZnO and TiO_2_, can absorb both visible and ultraviolet light, hence exhibiting the capacity to impede the development of microorganisms through redox reactions (Chakraborty et al. 2023).

Transition to nanocoating for multifunctional applications recently emerged as an economical and efficient technique for protection against biofouling and corrosion. However, traditional coating utilises single-function anticorrosion or anti-biofouling. In the anticorrosion coating, the primary objective is to prevent corrosion; however, the antifouling protection is ignored. These limitations focus on only one specific role; hence, it is crucial to incorporate multitudinous characteristics into a single coating. Therefore, integrating antifouling and anticorrosion characteristics within a single coating exhibits considerable potential for future applications. This multitudinous approach provides a more attractive option as it is an effective and economical solution for industries to resolve two major challenges in a single solution.

This review summarises research advancements made in selected functional coating materials incorporated with nanotechnology for improved characteristics and performance against biofouling and corrosion. However, despite the extensive research conducted on coatings in earlier decades, there is a lack of comprehensive documentation about the diverse applications of nano coating that have lately emerged. In contrast to prior scholarly works, this study introduces nanotechnological progress in the field of multifunctional coatings, focusing on the recent emergence of inorganic and organic hybrid substrates. The integrated multifunctional nanocoating is nascent and gaining the scientific community’s attention. As far as we know, only one review investigation has focused on integrated multifunctional antifouling and anticorrosion coating (Jin et al. 2022). This review comprehensively provided an overview of the integrated multifunctional coating based on polymers and other materials. However, this review investigation lacks a comprehensive study on nanometal oxide composites based on integrated multifunctional antifouling and anticorrosion coating. Thus, a review focusing on the prospectives of nanometal oxide composites will capture the attention of anticorrosion and fouling communities and instigate the advancement of integrated multifunctional antifouling and anticorrosion coating.

Today, nanocoating has ascended as the best coating and literally as the optimum top coating in its various domains of applications; therefore, it is imperative to have an in-depth investigation of multifunctional coating aspects. The initial session of this review article discusses the fundamental concepts and mechanisms of biofouling and corrosion, which will provide an overview of these concepts. The prospectus of integrated nanometal oxide composites on multifunctional coating is discussed elaborately, followed by challenges and future perspectives. This review gives the current status and will support and assist the scientific community in understanding fundamental concepts, prospectus, and challenges of integrated multifunctional antifouling and anticorrosion nanocomposite coating. It will also further motivate the introduction of novel innovative concepts to overcome present drawbacks.

## Concept of corrosion and biofouling

### Corrosion mechanism

Marine corrosion is often attributed to many intricate causes that are classified into physical and chemical corrosion. For example, the movement and rotation of the ship’s propellor lead to cavitation corrosion. Similarly, the contact of material with micro and macro particles such as sand and liquid movement causes erosion corrosion (Zadeh and Zadeh 2023). In general, exposing the material to sea water and moving at high speeds causes abrasion, mechanical tear, and wear. Erosion corrosion occurs in areas where the protective layer has been eroded as a result of deterioration and mechanical tear. In addition to these physical corrosion types, electrochemical or chemical reactions are further causes of corrosion. Furthermore, the surface of saltwater possesses an ample amount of carbon dioxide and oxygen that may effectively come into touch with the metal surface due to the combined effects of wave movement and seawater scouring (Wu et al. 2022). This, in turn, enhances the electrochemical and chemical interactions occurring between the metal and seawater. The existence of oxygen contributes to the oxidation of the metal surface, which causes in the development of a thin oxide layer which, commonly referred to as chemical corrosion (Daniel et al. 2023; Wang et al. 2023). Electrons get detached from the metal surface at the anode, resulting ionisation. Simultaneously, the reaction between oxygen and water molecules results to the formation of hydroxide ions. The metal and hydroxide ions react to produce metal hydroxide, which eventually transforms into metal oxide. Consequently, the metal on the surface is eliminated in the form of its corresponding oxide. The corrosion resistance of the material is contingent upon the specific environmental conditions to which it is exposed. Certain types of metal exhibit corrosion resistance in specific environments, whereas they may be susceptible to corrosion in different environments. For instance, aluminium exhibits accelerated corrosion in a diluted soda solution, but it has strong resistance in other types of solutions. The stainless steel AISI 316, composed of 18% chromium, 10% nickel, and 2% molybdenum, is known for its higher corrosion resistance compared to AISI 304, which has 18% chromium and 8% nickel. Therefore, AISI 316 is chosen over AISI 304 for safety purposes. Nevertheless, when exposed to nitric acid and several other acids and oxidising solutions, it is evident that 316 is less resistant to corrosion compared to 304.

**Fig. 2:**
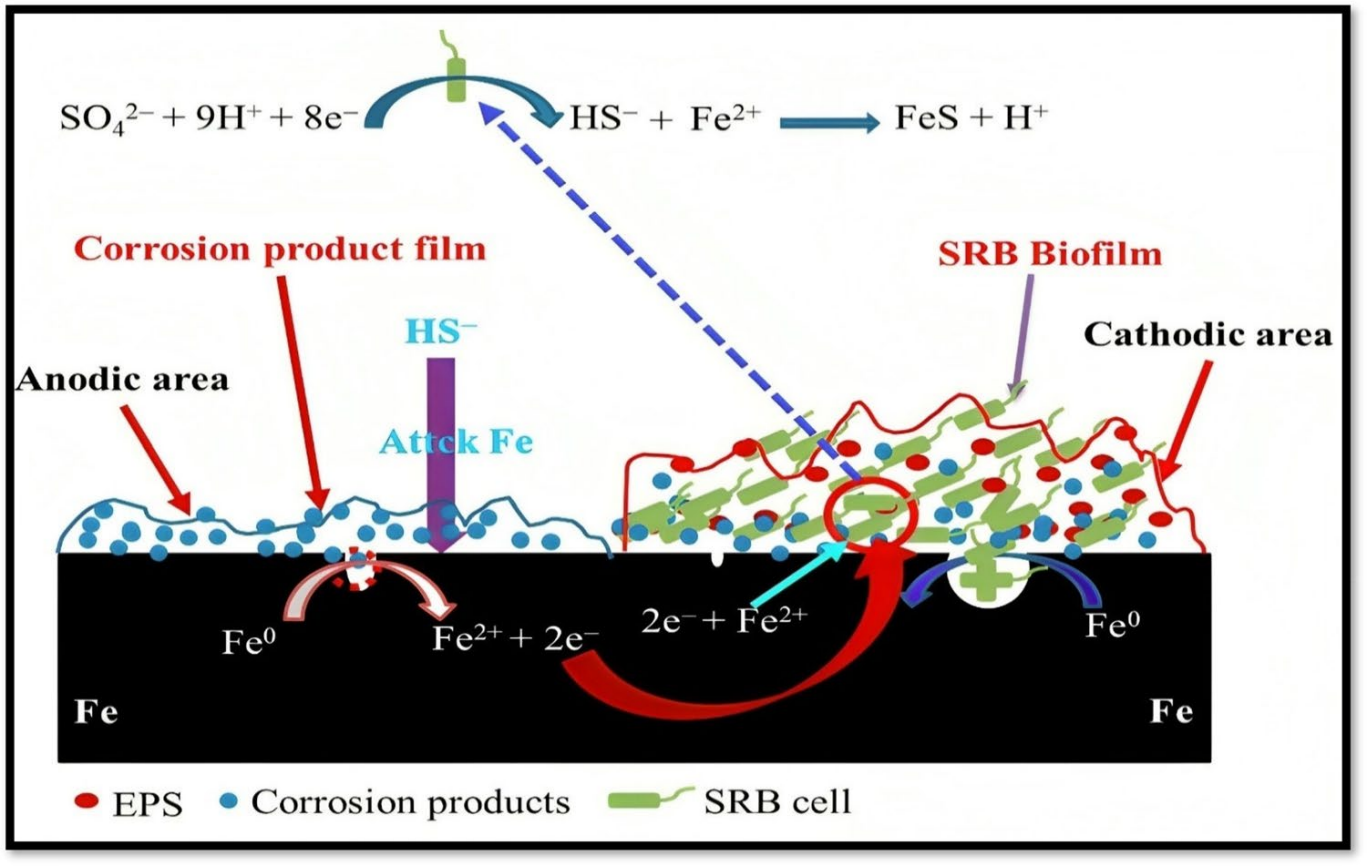
A mechanistic model illustrating galvanic corrosion behaviour of sulphate reducing *Desulfotomaculum nigrificans* biofilm-covered and uncovered carbon steel, where EPS refers to extracellular polymeric substances. Initially the specimen in the abiotic solution is as the anode, and some local anodic sites can be found on the specimens’ surface in the abiotic and SRB (sulphate-reducing bacteria)-containing test solution (Liu et al. 2020)

In addition to the primary corrosion process, microorganisms could contribute to corrosion (Li et al. 2023b). Microbiologically induced corrosion occurs when microbes modify the metal surface physically or chemically, making it more vulnerable to corrosion (Li et al. 2024). The main microorganisms that cause corrosion are fungi and bacteria. The most favourable temperature environment for microorganisms is from 15 to 45 °C at a pH level in the range of 6–8 (Saleh and Hassan 2024). The microbiological interactions with materials can occur either directly or indirectly. Figure 2 illustrates the schematic mechanisms of the microbial-induced corrosion mechanism occurring on the iron surface. Once the microorganisms are attached to the metal surface, they require energy for survival and growth. When there is a lack of electron donors, the sessile cells at the bottom of the microbial biofilm will search for alternative electron donors for sulphate oxidation in its energy production (Xu and Gu 2014). As an alternative source of electron donors, the sessile cells utilise the elemental iron present in the metal surface as its abundantly available leads to corrosion. The microorganisms interlink with the metal surface through their metabolism in direct interactions. In contrast, indirect interactions through anaerobic and aerobic mechanisms involve microbial generation of corrosive reactants such as inorganic and organic acids as well as sulfides, phosphides, and ammonia (Puentes-Cala et al. 2022).

In addition, microbiological-induced corrosion is further classified based on the presence and absence of oxygen in the environment. An environment that lacks free oxygen is referred to as “anaerobic.” Water reduction is important to provide the cathodic current required to sustain and perpetuate the corrosion process. This indicates that bacteria play an integral part in iron corrosion by extracting and using hydrogen present in the cathode regions of the metal to convert sulphate into sulphide by reduction. An environment can be classified as “aerobic” when the dissolved oxygen content is sufficiently high to sustain the corrosion process without the need for water reduction. Aerobic bacteria thrive by incorporating carbon dioxide through the utilisation of energy obtained from the oxidation of sulphur to form sulphite, which supports their growth and survival. Because anaerobic bacteria may reduce sulphate directly on the metal surface, pitting corrosion can occur in specific areas. Typically, surface microbes induce local variations in dissolved oxygen levels, ions, redox potential, pH, and conductivity (Amendola and Acharjee 2022). Localised evolution can intensify the electrochemical and chemical interactions between microbes, medium, and metals, resulting in surface corrosion and subsequent failure.

### Biofouling mechanism

**Fig. 3:**
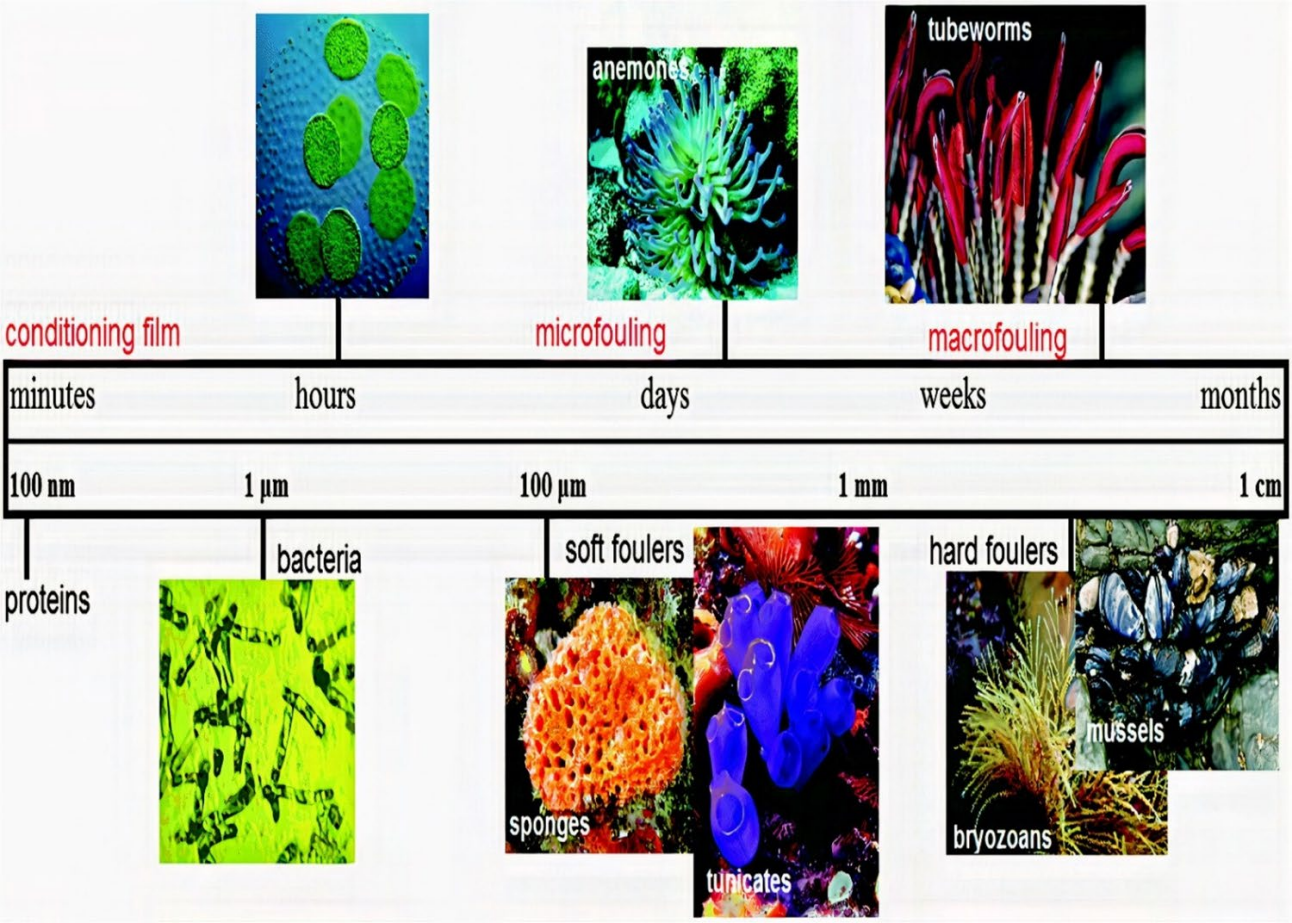
Various phases of marine biofouling: how they change over time and how rough they get (Nurioglu et al. 2015)

The term biofouling is employed to depict organisms attachment on submerged surfaces due to intermolecular or mechanical interactions (Karyani et al. 2023). When a surface is submerged in ocean water, the proteins, polysaccharides, organic and inorganic macromolecules instantly adsorb to the surface, forming a conditioning film (Shineh et al. 2023). After a certain period, which might range from minutes to hours, microorganisms begin to adhere to the conditioning film, as shown in Fig. 3. Antifouling research identifies the prevailing microorganisms as diatoms, algae spores, and bacteria (Romeu and Mergulhão 2023). Diatoms and bacteria can synthesise extracellular polymeric substances (EPS) to promote growth and defend against detrimental agents (Costanzo et al. 2023). Over time, these processes culminate in developing a compact biofilm on the substrate. Forming biofilm will attract and aid the growth of principal species associated with biological deposits, such as algae, crinoids, tubeworms, and mussels. Numerous environmental factors influencing biofouling growth include pressure, pH, substratum composition, sunlight, dissolved oxygen, nutrient availability, salinity, water temperature, and oceanic environment, which further cause temporal and spatial variations in the attachment depending on the location and depth (Saini et al. 2023). Table 2 lists various techniques employed for assessing microorganisms’ growth and activity attached to the surface.

**Table 2:** Various techniques employed for assessing microorganisms’ growth and activity attached to the surface

Biofouling monitoring tests	Description	Advantage	Disadvantages
Light microscopy	• Basic microscopic technique • The interaction between light and microorganisms in the presence of specific stains is analysed • Preliminary analysis to study the initial stage of biofilm growth	• Easy to use and quick analysis	• Poor resolution • Accuracy
Epiflourescence microscopy	• The presence of biofilm is detected by the light emitted from the activated molecules within its structure • Subjecting the biofilm to visible or ultraviolet light of a specific wavelength causes some biomolecules in the biofilm matrix to absorb and then emit light with a longer wavelength • Prior to epifluorescence microscopic analysis, biofilm is treated with fluorochrome stains for staining	• Capable of exploring the physiological functions of living organisms • Two-dimensional image analysis • Estimate the total number of cells and the behaviors of microorganisms • Rapid analysis	• Not suitable for all organisms • Low resolution • Not able to assess biofilm depth
Electron microscopy	• Transmission and scanning electron microscopy • High magnification power to identify constituents of biofilm	• High resolution • Cross section details • Spatial distribution of microorganisms	• Complexity of sample preparation for analysis • Not able to study in situ and in vivo • Slow analysis process
Confocal laser scanning microscopy	• Laser light source employed • Deeply penetrates the biofilm to activate certain components • Detected by photomultiplier tubes further converts to images	• Three-dimensional images for better analysis and investigation • Able to identify gene expression in addition to metabolic activity and growth • Study physio, chemical, and biological aspects	• Slow scanning • Overlapping of fluorescence signals • Unsuitable in thick and opaque biofilm
Atomic force microscopy	• Provide detailed data on the topography of biofilm surfaces at the atomic level	• Roughness and height of micororganisms attached can be estimated • High resolution • Suitable in vivo studies	• Sample dehydration during analysis
X-ray microscopy	• To study the connections between biofilm and metallic elements • Mapping the positioning of ligands and the allocation of metals and macromolecules within the biofilm matrix	• High Resolution • Easy sample preparation for analysis • Maintains hydration	• Not suitable for thick biofilm
Differential interference contrast microscopy	• Used for investigating opaque biofilms • Biofilm surveillance on corroded surfaces is particularly important, as this microscopic method can visualize curved biofilms	• Three dimensional images	• Expensive • Fragile • Heat sensitive
Environmental scanning electron microscopy	• It allows hydrated biofilms for the analysis • Spatial distribution and biofilm structure can be observed,	• High resolution	• Not suitable for in vivo study
Digital time-lapse microscopy	• Real time monitoring able to study microbial adhesion under different environmental conditions • Able to measure cell growth, death, and detachment of organisms	• Realtime monitoring	• Not suitable for large areas
Fourier transform-infrared (FT-IR) spectroscopy	• Used for characterisation study of biofilm attached on the surface • Using infrared source to determine specific functional groups • Determine physiological status of the microorganisms	• Fast analysis • Accurate • Less volume for analysis	• Not suitable for thick biofilm • Need of update library of all microorganisms
Fluorometry techniques	• Able to monitor the activity of microorganisms • Using ultraviolet or visible light source to get quantitative and qualitative information regarding biofilm constituents	• Fast analysis	• Restricted to laboratory and industries studies • Only specific microorganisms
Photoacoustic spectroscopy	• Utilises light and sound source for analysing attached biofilm • Infrared or laser light source used • Able to measure the depth of biofilm • Detect growth of organism	• Depth of biofilm assessment	• Less resolution

### Multifunctional coating strategy

The biofouling and corrosion have some similarities and differences. The main similarity is that in the initial phase, biofouling and microbial-induced corrosion are initiated by biofilm formation on the material surface caused by microorganisms (Garibay-Valdez et al. 2023). The fundamental design concept of multifunctional coating is the integration of anticorrosion and antifouling components into a single coating system. The purpose of this review is to present an extensive summary of the latest developments in the formulation and efficacy of nanocoatings that incorporate active components with the specific objective of preventing fouling and corrosion. This session introduces and discusses the recent development of nanocoating-based multifunctional coating.

### Multifunctional coatings based on graphene

Graphene is a two-dimensional nanomaterial comprising carbon atoms that are sp^2^ hybridised and interconnected in a hexagonal lattice structure (Li et al. 2023a). Graphene demonstrates remarkable electrical, mechanical, optical, and antibacterial characteristics, making it a effective addition to coatings to enhance their mechanical and antifouling attributes (Aissou et al. 2023). In addition to its superior thermal stability, chemical inertness, and shielding impact on liquid and gas, graphene has also been utilised in applications to prevent corrosion. Hence, incorporating graphene oxide with various metal oxides has gained wide attention from the scientific community in recent years. Ziyang Zhou and team studied on zinc oxide- 2-methylimidazole metal–organic framework wrapped with graphene oxide (GO) protective coating against fouling and corrosion (Zhou et al. 2023c). Graphene oxide nanosheets inherit oxygen functional group which utilises as nanofillers as advanced anticorrosion nanofillers in epoxy coatings. In addition, graphene oxide entails antibacterial characteristics that cause oxidative stress on microorganisms and limit their growth. This investigation aims to exploit the beneficial attributes of GO nanosheets and ZIF-8 (zinc oxide- 2-methylimidazole) by incorporating them into GO@ZIF-8 nanohybrids with silane moieties enveloping their surface. Zeolitic imidazole framework-8 (ZIF-8) is used as a functional filler and curing agent to prepare epoxy nanocomposites. The imidazole group on the surface of the ZIF-8 initiates epoxy curing, resulting in covalent bonding between the ZIF-8 crystals and epoxy matrix. This is done to enhance the durability of aqueous epoxy-PDMS coatings when exposed to corrosive substances and fouling environments. Furthermore, the silanes act as a protective coating against moisture and as surface modifiers as well as coupling agents due to anti-corrosion, environmentally friendly, and adhesion characteristics. The adhesion characteristics between film and metal function as a protective layer to prevent the diffusion of oxygen and corrosive ions. The -OH group is responsible for the hydrophilicity of surfaces, and therefore, substituting them with Si-R (where R = alkyl or aryl) groups prevents water adsorption, resulting in a hydrophobic surface. The *Pseudomonas* sp. bacterial anti-fouling properties assessed in the oceanic environment exhibited a reduced attachment of bacteria and marine species to the coating. In addition, the corrosion studies unequivocally demonstrate a corrosion protection resistance of 3.47 × 10^9^ Ω.cm^2^.

Ziyang Zhou further investigated the distinct contributions of graphene oxide and nanohybrids (Zhou et al. 2022b). According to the findings, the coating with evenly distributed F-GO@ZnO QDs (amino-silane functionalised ZnO quantum dots on graphene oxide) on its fracture surface has the potential to offer excellent corrosion resistance and barrier properties for steel substrates. ZnO quantum dots enhance the composite’s mechanical and chemical interactions, improving antibacterial and anti-corrosion attributes. It has excellent dispersion with no aggregations, high hardness, a low refractive index, and hydrophobic enhancement. F-GO@ZnO QDs exhibit superior water contact angle (88.5° ± 1.4°) and substrate adhesion strength (8.1 ± 0.4 MPa). Nanocomposite coatings exhibiting exceptional adherence to the metallic substrate are highly resistant to delamination and effectively maintain their protective properties against electrochemical corrosion reactions. Additionally, the coating maintains a pristine and sleek surface even after being submerged in saltwater for 30 days, demonstrating its effective anti-adhesion properties. After 60 days of immersion, biological adhesion has formed on the surfaces of both coatings.

**Fig. 4:**
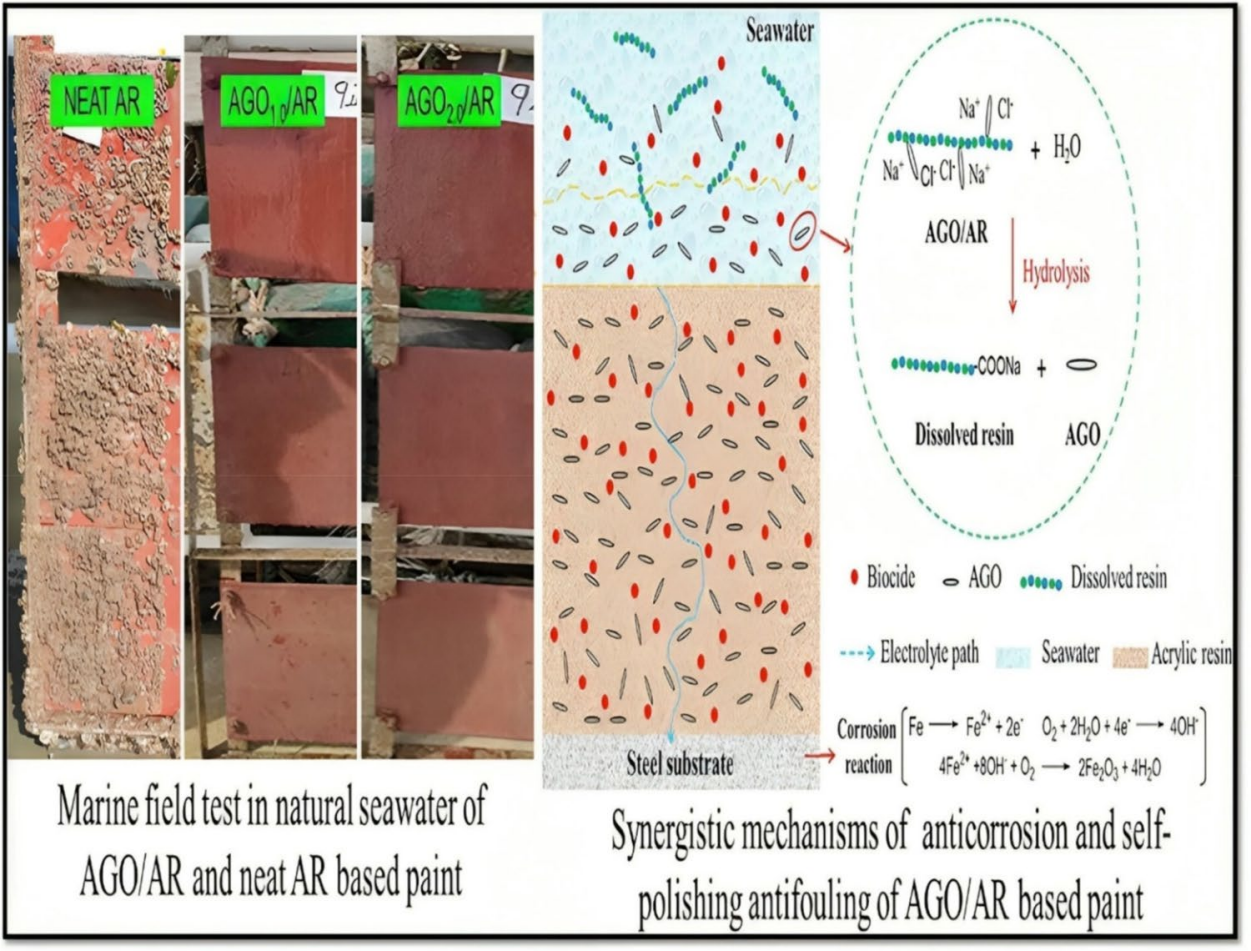
Mechanism and marine test results of AGO (Acrylic acid modified graphene oxide)/AR (acrylate) composite for multifunctional applications (Li et al. 2022b)

Yue Li (Li et al. 2022b) studied the synergistic mechanisms of anticorrosion and antifouling characteristics of acrylic acid-modified graphene oxide (AGO)/acrylate (AR) composites. Combining graphene oxide with acrylate composite demonstrated corrosion resistance and antifouling characteristics with a corrosion current density of 9.468 × 10^−8^ A.cm^−2^, whereas the bare steel noticed a corrosion current density of 4.29 × 10^−6^ A.cm^−2^. Furthermore, the composite also exhibited self-polishing antifouling capability in the ocean environment. After being subjected to a 360-h salt spray test, the AGO/AR surface showed minor damage, with only minor rust spots and no signs of corrosion widening. Therefore, the salt spray test outcomes confirm that AGO may improve the anticorrosion attributes of acrylic resin. In addition, the AGO/AR coatings were investigated for antifouling characteristics by immersion in the sea for six months, as shown in Fig. 4. Zihao He and his team studied graphene oxide/silane composite coating and its prospect as a corrosion and biofouling resistance coating (He et al. 2022). This study utilises the properties of 2D graphene oxide and silane molecules to develop an environmentally friendly and simple electrodeposition method for creating a superhydrophobic coating without fluoride. The coating has a remarkable protection efficacy of 99.98 ± 0.10% and remarkably declines the corrosion current density by about four orders of magnitude contrasted to the uncoated aluminium alloy. The compact GO sheets smooth out the composite coating’s surface and lessen the spots where adhesion occurs, preventing biofouling organism’s attachment.

Geetisubhra Jena and colleagues developed graphene oxide-nano SiO_2_-polydimethylsiloxane composite coating (GSP) on carbon steel for maritime applications focusing on corrosion and fouling resistance (Jena et al. 2021). It was clear from the galvanic coupling tests that adding nano-SiO_2_ to GO makes it more resistant to rusting. The new hybrid layer is very good at remediating bacteria and biofouling because it has a toxic effect on microorganisms from GO that doesn't let bacteria stick to the PDMS surface. The impedance values observed for GSP are around 10^9^ Ω.cm^2^ at lower frequencies, and the phase angle value is close to -90°. These results validate the exceptional coating qualities compared to uncoated steel surfaces. The GSP coating had polarisation resistance and charge transfer resistance values of six orders of magnitude greater than the uncoated carbon steel. The galvanic coupling investigations evidently show that the addition of nano-SiO_2_ to GO leads to an improvement in polarisation resistance (4.88 × 10^7^ Ω.cm^2^) compared to the uncoated sample (2.34 × 10 Ω.cm^2^).

### Multifunctional nanocoating based on metal oxides

The use of nanoparticles in conventional coatings has the potential to significantly improve a wide range of desired characteristics. Nanomaterial’s intrinsic antibacterial and photocatalytic activity can potentially restrict the development of organisms that cause fouling. Additionally, introducing nanoparticles can potentially modify the wettability and surface roughness of the coatings, reducing the adhesion strength of microbes that cause fouling.

#### Zinc oxide (ZnO)

Biocompatible zinc oxide (ZnO) nanoparticles exhibit photocatalytic and photooxidative activities towards microorganisms and have significant potential for use in antibiofouling and anticorrosion coatings (Zhou et al. 2023b). Xiaofan Zhai and their team recently synthesised ZnO/Zn nanopillar films through electrodeposition for marine antifouling and anticorrosion (Zhai et al. 2020). In this research, capsaicin was introduced into an alkaline electrolyte to develop the growth of ZnO nanopillars. ZnO/Zn nanopillar films were produced using a single cathodic electrodeposition by adding capsaicin to the electrolyte. The electrolyte’s capsaicin content was absorbed into the electrodepositing surface via the interaction with the functional –NH– groups in the amide bond. The resulting films of ZnO/Zn nanopillars exhibited significant antibacterial activities against suspended *Escherichia coli* solutions, with relatively limited bacterial penetration. This suggests a potential use in marine antifouling. The electrochemical assessment demonstrated that the ZnO/Zn nanopillar films (*R*_ct_ 1.15 Ω·cm^2^) produced by capsaicin displayed a significant improvement in their ability to resist corrosion when exposed to a media containing sulphate-reducing bacteria (SRB) compared to carbon steel (*R*_ct_ 1.59 Ω·cm^2^).

**Fig. 5:**
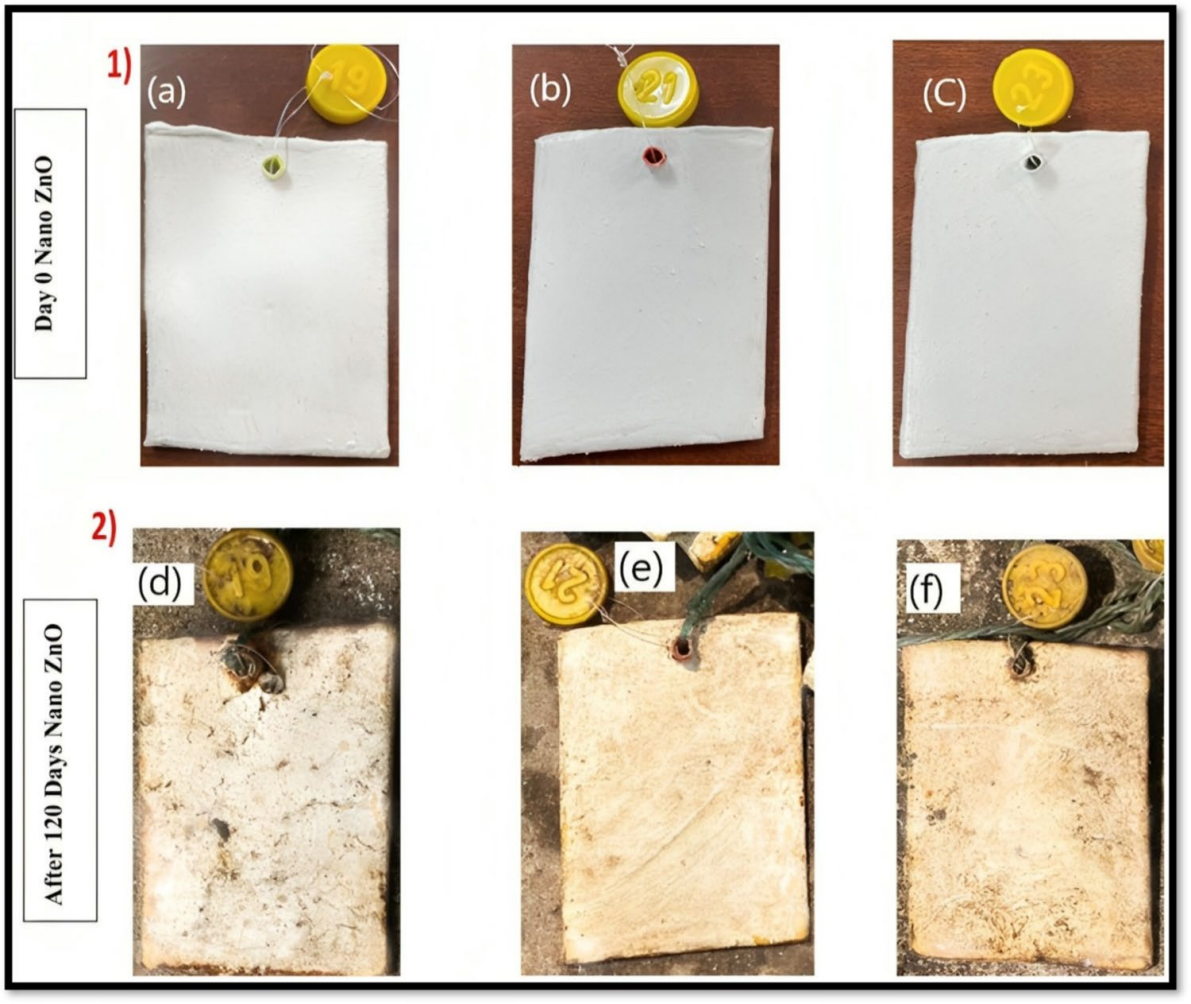
ZnO multifunctional paint formulations coated to steel specimens and submerged in the ocean for a duration of 120 days (**a**, **d** 0.25 gm; **b**, **e** 0.5 gm; **c**, **f** 1gm of ZnO) (Mohan et al. 2022)

Mohan and colleagues further investigated the prospective of ZnO metal oxide paint formulations (Mohan et al. 2022). The primary goal of this research is to create paint formulas that are resistant to fouling and corrosion, specifically for maritime structures and boats, by including nanoparticles as functional additives. The process included incorporating ZnO nanoparticles into linseed alkyd resin and combining them with thickener, soya lecithin, and mineral turpentine oil to create nanometal oxide paints. ZnO antifouling paint formulations coated to steel specimens and submerged in the ocean for a duration of 120 days. Notably, ZnO nano paint exhibits superior anti-fouling and anti-corrosion characteristics with less adhesion and minor corrosion spots, as shown in Fig. 5.

Aurkalam et al. investigated perfluorodecyltrichlorosilane-based poly(dimethylsiloxane)-ZnO nanocomposite coating with anti-fouling and anticorrosion effectiveness (Arukalam et al. 2016). Perfluorodecyltrichlorosilane enhanced the surface characteristics of the coating and has improved by reducing surface energy and minimising adhesion strength. The coating has a minimal adhesion strength of 1.88 MPa and a low surface energy of 26.59mN/m. The research team claimed that the coating exhibits exceptional antifouling properties, which may be attributed to the association between the surface chemistry of the coating and the adhesivity of biomolecules. In addition, the corrosion investigations demonstrated better corrosion protection (*R*_ct_ = 2.40E + 05 Ω·cm^2^) compared to uncoated Q235 steel (*R*_ct_ = 1075 Ω·cm^2^). Elmira Velayi and colleagues synthesised hybrid ZnO/CuO nanopowders for corrosion-resistant and water-repellent coatings (Velayi and Norouzbeigi 2019). The chemical precipitation techniques were employed to synthesise ZnO–CuO hybrid nanoparticles. The composite obtained exhibited outstanding superhydrophobic characteristics, with WCA (water contact angle) of 162.6° ± 1° and a contact angle hysteresis of less than 5°. The coated stainless-steel mesh produced with the optimal sample exhibited distinctive anti-corrosion and self-cleaning characteristics (*I*_*corr*_ = 4.58 × 10^−9^ Acm^−2^) and stability, particularly under ambient atmospheric conditions for 7 days compared to uncoated steel mesh (*I*_*corr*_ = 3 × 10^−8^ Acm^−2^).

Huimin Zhou and colleagues fabricated ZnO/epoxy resin superhydrophobic coating and investigated its suitability as corrosion and antifouling resistant (Zhou et al. 2019). This research introduces a novel approach inspired by the grass growth process to address the issues of poor wear resistance, adhesion, and poor corrosion resistance commonly found in superhydrophobic coatings. The critical strategy involves precise control of the ratio between epoxy resin and ZnO seeds, ensuring that the ZnO seeds are not fully coated. A cluster-like ZnO coating, consisting of interlinked ZnO rods, was created based on this approach. Inaddtion functionalisation with stearic acid, the coating exhibited superhydrophobic characteristic with contact angle of 163°. The coating demonstrated exceptional corrosion resistance imputed to the barrier provided by both the epoxy resin/ZnO seeds and the cluster-like ZnO. Meiling Zhang et al. synthesised lubricant-infused coating by double-layer ZnO on aluminium and studied its anti-corrosion and fouling characteristics (Zhang et al. 2018). The hydrothermal and sol–gel techniques were employed to synthesis double-layer zinc oxide on an aluminium substrate. An aluminium sheet, which had been coated with zinc oxide, was immersed in a lubricating fluid for a duration of 2 h. This immersion evolved in the development of slippery double-layer ZnO surface. In comparison with untreated sheets, the composite exhibited excellent corrosion resistance and antifouling attributes. These characteristics were evident in the longer run, indicating their excellent suitability for numerous applications.

Similar investigations were carried out by Milad Abdolahzadeh Saffar and their team (Saffar et al. 2022). The research team had successfully developed a superhydrophobic ZnO thin film modified by stearic acid on the copper substrate for corrosion and fouling protection. Sol–gel technique were employed to synthesis thin ZnO layer directly onto the copper substrate. Subsequently stearic acid was employed to modify the film. The WCA on the copper and zinc oxide-coated copper specimens elevated to 155° during the experiment. Similarly, the corrosion current densities declined from 1.31 to 2.7 × 10^−3^ μA/cm^2^, respectively. Furthermore, during the biofouling investigation, no adhesion of organisms was noticed in the ZnO thin film. Huilian Zhou and colleagues developed superhydrophobic nano-structured ZnO nanosheets (Zhou et al. 2022a). Cyclic voltammetric electrodeposition technique utilised to develop nanostructured zinc oxide nanosheets. The surface energy was further reduced by using Triethoxy(octyl)silane. The fabricated superhydrophobic surface has a corrosion inhibition rate of 99.83% compared to the untreated steel surface. The anti-fouling efficacy and photoelectric analysis demonstrate that the created superhydrophobic surface can withstand and break down contaminants by exposure to light, improvising the adaptability of the superhydrophobic surface suitable as the antifouling coating. The findings indicate that ZnO has excellent potential for employing antifouling and anticorrosion applications.

#### Copper oxide (Cu_2_O)

**Fig. 6:**
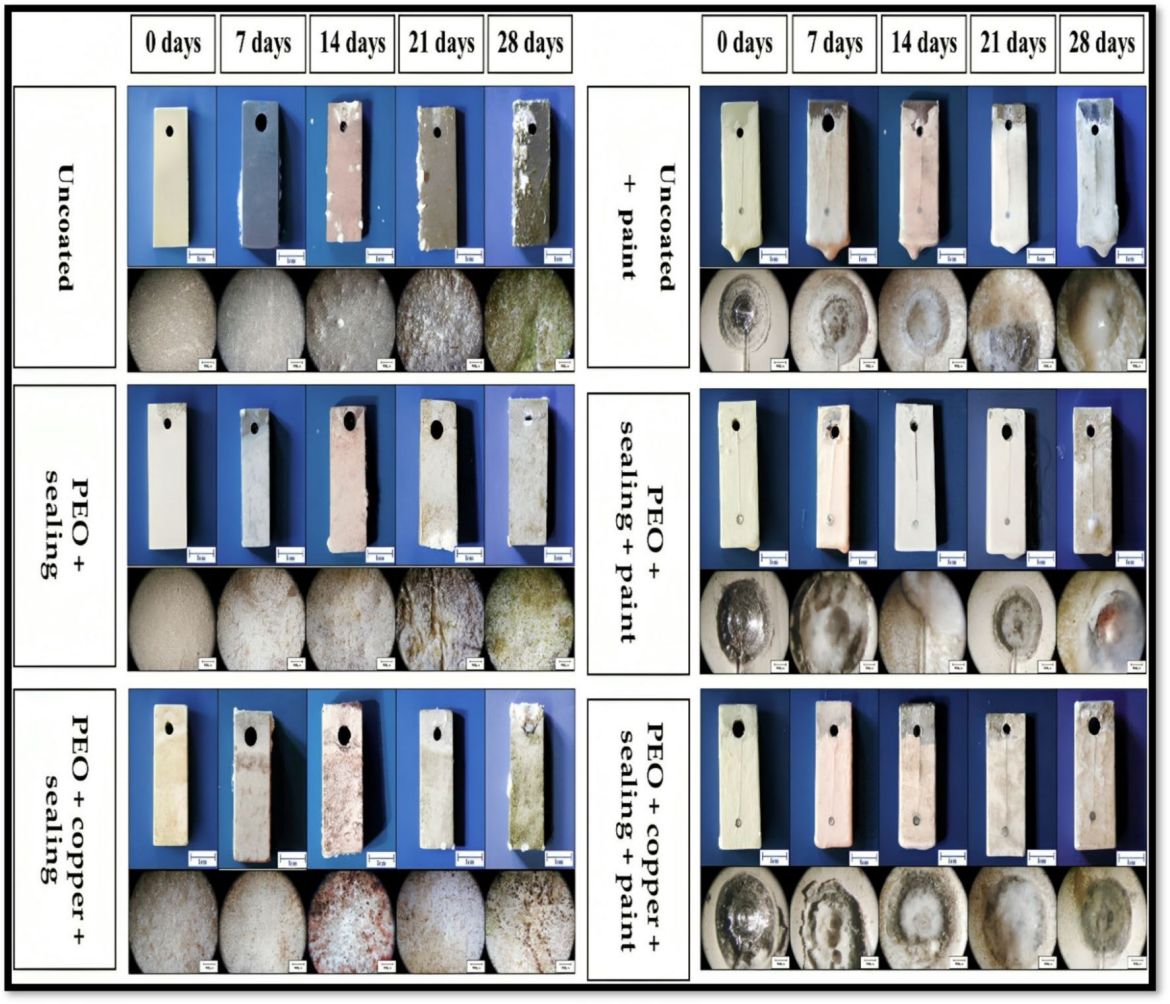
Marine environment testing of PEO-coated samples with and without copper coatings (Cerchier et al. 2020)

Copper oxide (Cu_2_O) is presently the most extensively used antifouling biocide in maritime paints. Copper oxide is a suitable candidate for corrosion and fouling resistance applications. It has been widely studied over time, but less work has been carried out to investigate it for multifunctional antifouling and anticorrosion applications. Cu-Co composite coating was studied as a potential candidate for antifouling and corrosion resistance (Zhang et al. 2023). Based on the findings, it can be summarised that the superhydrophobic coating demonstrates better minimal self-corrosion current density and a stronger charge transfer resistance. The superhydrophobic sample interface has a micro-nano rough structure with minimal surface energy, effectively capturing air and creating an air layer. This air layer minimises contact within the corrosive medium and the surface. In addition to thermal durability, the superhydrophobic coating exhibits self-cleaning and mechanical characteristics. The corrosion current density of 5.2227 × 10^−7^ A·cm^−2^ was measured and compared with B10 Cu Ni alloy 8.2259 × 10^−5^ A·cm^−2^. In addition, the average WCA was 162.2 ± 1°, and the roll-off angle was less than 2.1°. Chasse and team investigated copper-based protective coatings against fouling and corrosion (Chasse et al. 2020). In the studies, the copper oxide demonstrates excellent protection against fouling and corrosion during its 90 days of natural seawater exposure. Polyethylene glycol-based copper oxide is utilised as a propitious antifouling and anticorrosive coating for maritime applications (Cerchier et al. 2020). Utilising a copper-containing PEO (polyethylene oxide) coating imparts excellent corrosion qualities and resistance to fouling to the specimen compared to uncoated aluminium alloy. Furthermore, using copper in the PEO reduces the growth of biofouling organisms, even when there are flaws in the paint. The tests conducted in saltwater demonstrated the corrosion resistance characteristic of PEO treatment compared to the untreated sample. After 14 days of submersion, the untreated samples exhibited a significant amount of corrosion products, clearly visible as white spots in the stereomicroscope images. No corrosion products were detected in the samples treated with PEO, even after a duration of four weeks of immersion, as exhibited in Fig. 6. PEO exhibits greater adhesion to the substrate, preventing corrosion, and the PEO coating prevents the blistering problem associated with alloy coating. Additionally, incorporating copper powder into PEO coating prevented fouling colonisation.

Yan Lv and team developed copper-based superhydrophobic coating and studied its fouling and corrosion behaviours (Lv and Liu 2019). The superhydrophobic coating that forms on the copper substrate surface exhibits a static contact angle (SCA) of up to 157° and a contact angle hysteresis of 4.2°. Compared to the exposed copper substrate surface, a superhydrophobic coating exhibits enhanced resistance to corrosion and anti-fouling characteristics. The corrosion current of the superhydrophobic coating on the copper substrate decreases to 6.8911 × 10^−6^ A cm^−2^ from 5.5577 × 10^−5^ A cm^−2^. In contrast, the corrosion potential rises to -0.1966 V from -0.2878 V. This implies that the superhydrophobic coating has a more robust corrosion protection characteristic than the bare copper substrate. The process involves interacting with copper atoms on the copper substrate and octadecanoic acid molecules, forming a long carbon chain structure on the copper surface. This structure imparts superhydrophobic characteristics to the copper surface. The fouling rate of the superhydrophobic coating on the copper substrate is merely 2.1833 g m^−2^ d^−1^ due to its superhydrophobic nature.

#### Silver oxide (Ag_2_O)

**Fig. 7:**
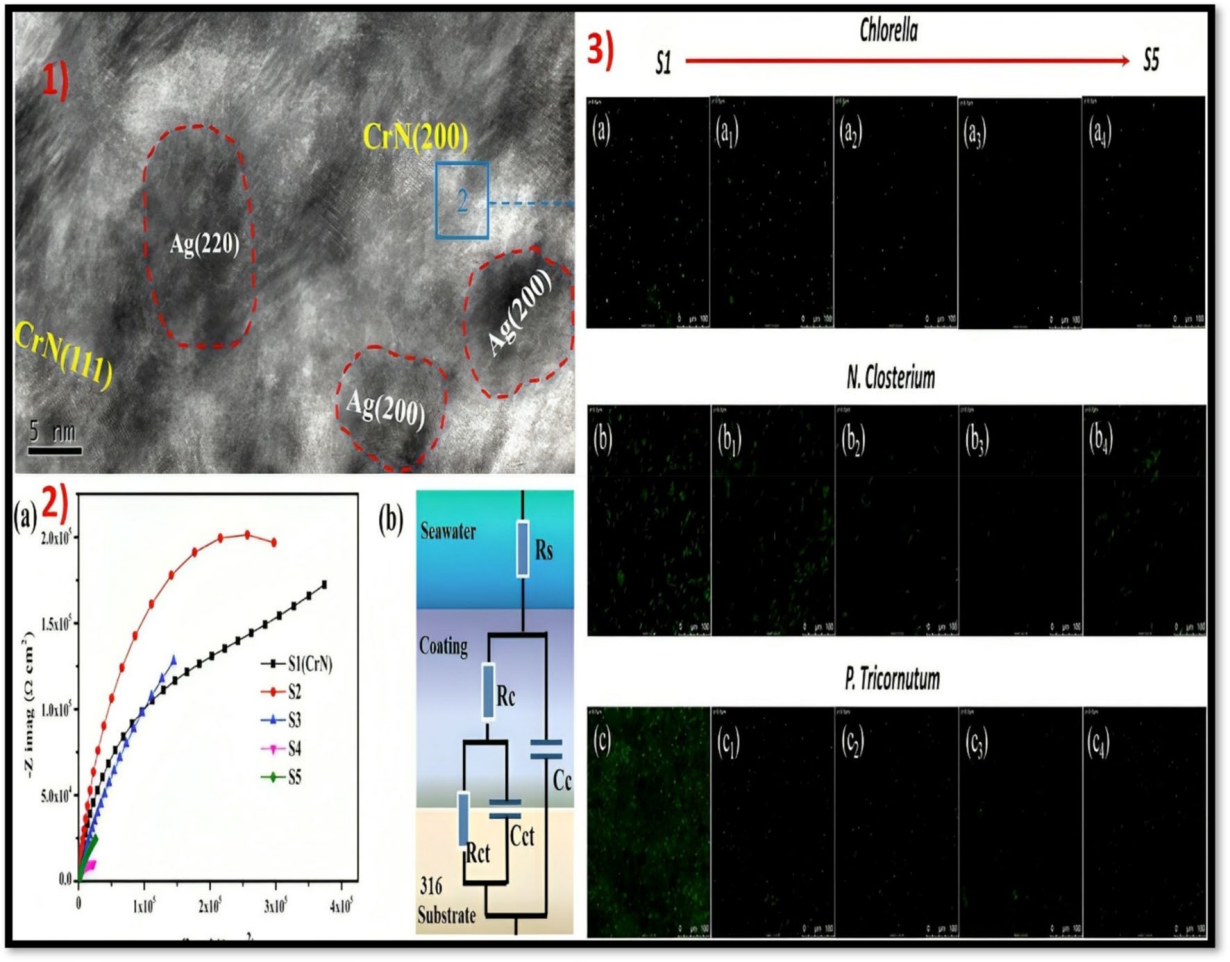
1) a—TEM image of silver-doped CrN coatings, 2) a—electrochemical anticorrosion investigation (S2-4.96, S3-8.06, S4-13.18, and S5-18.37; different weight percentages of Ag in composite), b—circuit model of composite; and 3) antifouling tests with various organisms (Cai et al. 2018)

Silver is one of the commonly employed materials for numerous applications such as health care, medicine, electronics, etc. Numerous studies have been reported on silver and its prospectus for antibacterial and microbial applications. Silver-doped CrN coatings deposited by magnetron sputtering were investigated for antifouling and corrosion resistance (Cai et al. 2018). The findings demonstrated that adding silver to the coatings deteriorated their ability to resist corrosion. The build-up of Ag in the layer decreased diffusion and release, thereby impeding the aggressive environment from reaching the underlying material and attenuating the rate of corrosion upon exposure to the corroding medium. This suggests that silver enhances the reactivity and facilitates the development of corrosion. However, a contrasting trend was seen in terms of anti-algae behaviour. The coatings with a silver concentration of 13 atomic percent exhibited exceptional anti-algae activities and superior antibacterial characteristics against *Bacillus subtilis* compared to *Escherichia coli*. The adhesion of *Chlorella*, *N. clostridium*, and *P. tricornutum* to the coated surface decreases by about 45%, 72%, and 64%, consecutively, as shown in Fig. 7.

Yujie Hu and colleagues recently synthesised Ag/Cu bimetallic nanoparticles utilising ginger extract (Hu et al. 2022). This work introduced a sustainable approach for producing Ag/Cu bimetallic nanoparticles as innovative substances that may prevent corrosion and function as biocides. The findings demonstrated that Ag/Cu bimetallic nanoparticles, synthesised from ginger extract at a concentration of 6.25 μg mL^−1^, effectively compromised the structural integrity of SRB (sulphate-reducing bacteria) cells and significantly decreased their adherence to the carbon steel surface by more than 65%. The corrosion rate of bimetallic nanoparticles exhibited a decline from 0.453 to 0.05 mm·a^−1^, whilst the adhesion was below 65% in comparison to the uncoated sample.

Similar investigation on the fabrication of silver nanoparticles on a Cu-substrate was conducted to study its photocatalytic activity, anti-corrosion, and superhydrophobicity (Khan et al. 2022). The silver doping enhanced the hydrophobic nature, which aids antifouling and self-cleaning characteristics. In addition, the composite exhibited enhanced corrosion resistance. *I*_corr_ and *E*_corr_ were measured at approximately − 0.155 V and 5.774 × 10^–9^ A cm^−2^, respectively. The corrosion potential and current density of Cu@Ag-SHS (SHS- superhydrophobic surface) exhibited a gradual decrease in value, which can be attributed to the fabrication of silver nanoparticles on the copper surfaces. Chen and team successfully fabricated superhydrophobic silver/graphene coatings through a one-step electrodeposition method (Chen et al. 2022). The presence of silver on graphene and cerium compounds material composite improvises antifouling and corrosion resistance characteristics. The composite coatings noted a contact angle (CA) of 154.1° whilst the sliding angle was as low as 1°. Furthermore, the coating maintains a resistivity of 0.494 Ω.

Yajun Deng and colleagues researched modifying the surface of silver halloysite nanotubes by grafting them with 3-aminopropyltriethoxysilane (APTES) and 2-furoyl chloride. This modification facilitated the interaction amongst silver ions and the nitrogen/oxygen atoms of the grafted chemical units through their active lone pair electrons and empty orbits (Deng et al. 2022). The silver functionalised halloysite nanotubes were incorporated into a new coating and chemically bound inside the composite polybenzoxazine structure. The coating with a high-density structure demonstrated exceptional antifouling properties, including effective bacterial eradication and prevention of adhesion. Furthermore, it exhibited outstanding resistance to corrosion when exposed to immersion and salt-spray conditions for a duration of 126 days. Ag/SiO_2_ core–shell nanoparticles were investigated for antimicrobial corrosion, which could also prevent the growth and adhesion of microbial fouling organisms (Le et al. 2010). The findings demonstrate a significantly reduced corrosion current (*I*_*t*_) of 3.03E − 08 A cm^−2^, proving its exceptional corrosion resistance compared to uncoated steel. The superior corrosion protection of Ag/SiO2 core–shell nanoparticles can be attributed to the dual action of silver’s antibacterial properties and the silica shell. Silver effectively inhibits antimicrobial corrosion, whilst the silica shell retains and slowly releases silver, slowing initial corrosion processes. Additionally, –Si–O–Si– skeleton present on silica particles with partial ionic character exhibits surface-active properties that consistently resist microbial and other organisms. The Ag/SiO_2_ nanoparticles possess a substantial active surface region and a core–shell structure, enabling the reduction of the load and improved control over leaching. This composite can be widely used in creating antibacterial coatings and paints to provide long-lasting protection and ecologically friendly coatings in the maritime sector.

#### Tungsten oxide (WO_2_)

Rubina Basheer and her colleagues created a tungsten-wetted titanium dioxide composite coating to control corrosion and biofouling that are impacted by microorganisms (Basheer et al. 2013). Significant corrosion was seen when zinc coating was submerged in experimental solutions whilst measuring the self-corrosion rate. This corrosion was primarily caused by a strong bacterial presence in the absence of W–TiO_2_ coating. A reduction in optical density demonstrated the improved antifouling activity of the W–TiO_2_ composite. The hot-dip galvanisation technique involved in the subsequent addition of the W–TiO_2_ composite exhibits high antifouling activity and thermal stability. Minimal corrosion rate was reported in seawater containing biofilm scrapings as compared to uncoated steel samples. Similarly, doping of tungsten disulphide into cobalt was investigated for corrosion and wear resistance with antifouling characteristics (Liu et al. 2023). The integrated WS_2_ boosts microhardness and begins as a physical hurdle to keep corrosive solutions out, giving the deposited Co-WS_2_ composite coating exceptional durability against wear and corrosion. Compared to a pure Co coating that has a weak ability to resist fouling, the Co-WS_2_ composite coating, with its evenly distributed integrated WS_2_, exhibits minimal surface energy and exceptional resistance to wear and corrosion. Composite coating deposited with WS_2_ obtains the maximal positive *E*_*corr*_ of − 496.4 mV, the lowest corrosion current density of 5.17 × 10^−6^ A·cm^−2^, charge transfer resistance of 1.08 × 10^4^ Ω cm^2^ and the minimal corrosion rate of 0.61 × 10^−1^ mm·year^−1^. Embedded within the coating, WS_2_ fills pores and cracks whilst disrupting corrosion paths. Additionally, it serves as a physical obstacle that hinders corrosive liquids from infiltrating the composite coatings. The inclusion of WS_2_ results in grain refinement, boosting the number of grain boundaries and reducing the cathode/anode surface ratio to combat localised corrosion. The composite coating exhibits a minimal surface energy and a much larger WCA of around 131.4°, almost double that of the cobalt coating. The reduced surface energy may efficiently inhibit the adhesion of fouling organisms.

#### Titanium dioxide (TiO_2_)

Titanium dioxide (TiO_2_) is widely favoured because of its affordability, exceptional reactivity, strong durability, and low toxicity (Rajaram et al. 2023). TiO_2_ nanoparticles are extensively used as photocatalytic due to its low bandgap. This photocatalytic effects can eliminate microbial organism causing fouling and corrosions. P.Sumalatha Devi and colleagues developed nano silica- titanium dioxide composite for multifunctional coating for marine applications (Devi et al. 2017). It has minimal surface energy and is resistant to fouling organisms without the need for biocides or other hazardous substances. The field exposure experiments demonstrate that the coating offers long-lasting protection against the build-up of fouling organisms and corrosion on the surface. Despite being continuously exposed to seawater for 3 months, no rust or blistering occurred on the covered area. In addition, only microfouling observed on the surface during the immersion tests.

Yahong Li and colleagues examined the electrodeposition of Ni-W/TiO_2_ composite coatings on Q345 pipeline steel, their corrosion resistance, and their ability to prevent fouling in simulated oilfield wastewater (Li and Zhu 2022). As the percentage of TiO_2_ doping into the composite inclines, the coating characteristics also increase. This diminishes the activation energy required for crystal nucleation and promotes nucleation, resulting in a more compact surface for the composite coating. The Ni-W/TiO_2_ composite coating, developed with a TiO_2_ particle concentration of 4 g/L, exhibits a strong and compact surface with the highest TiO_2_ particle content of 3.90%. It also has a minimal surface free energy of 25.48 mN/m and a maximum contact angle of 120° with the highest charge transfer resistance of 6.4 × 10^3^ Ω cm^2^ compared to Q345 pipeline steel (2.3 × 10^3^ Ω cm^2^).

**Fig. 8:**
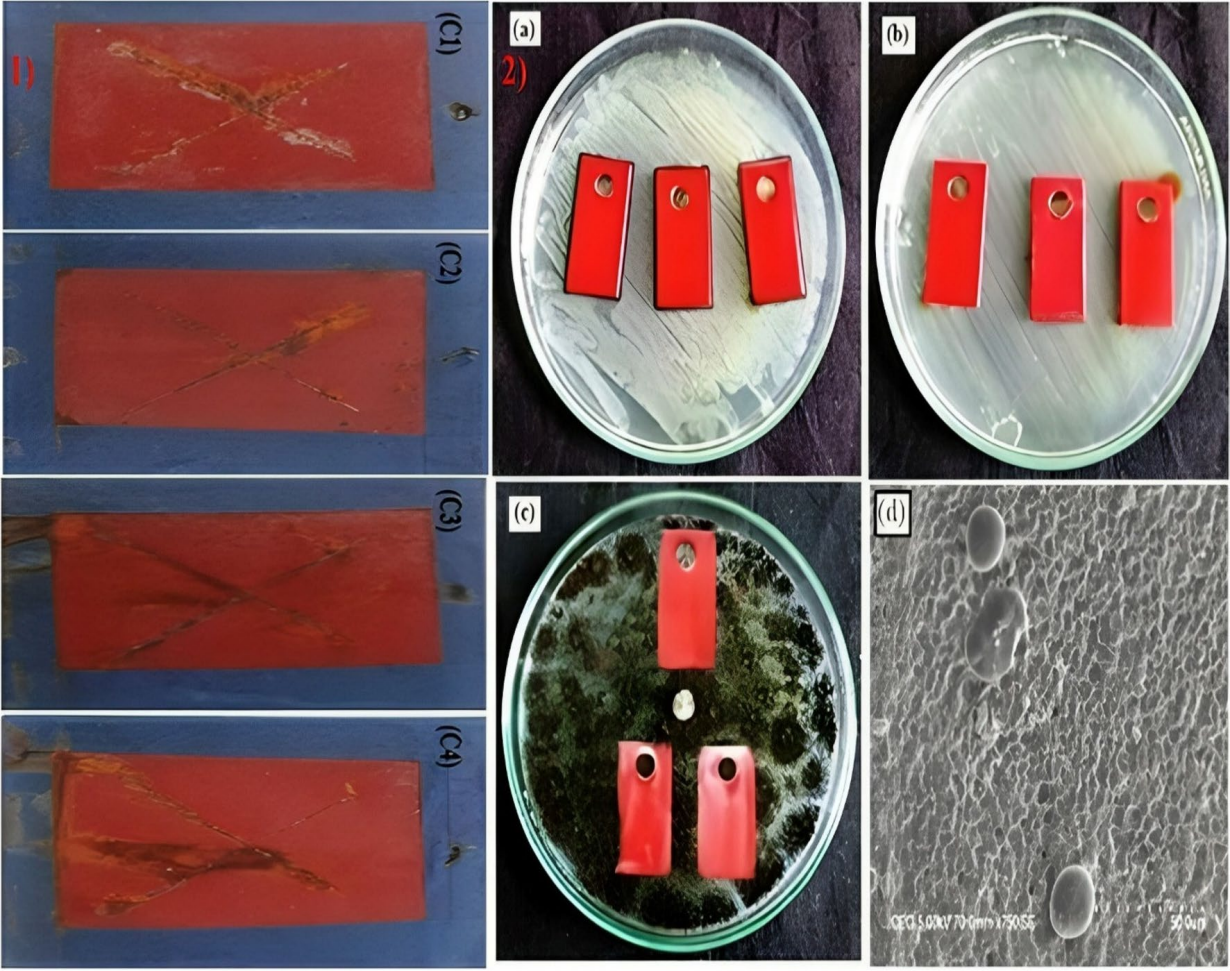
1) Salt-spray exposed TiO_2_–APTES–DGEBA coating (C1-1%, C2-3%, C3-5%, and C4-7% of TiO_2_) 2) microbial testing of **a** *S. aureus*, **b** *P. aeruginosa*, **c** *A. niger*, and **d** scanning electron microscopy image of composite after *S. aureus* exposure (Saravanan et al. 2016)

TiO_2_–APTES–DGEBA (3-Aminopropyltriethoxysilane) nanohybrid composite coating was investigated for corrosion and fouling resistance, as exhibited in Fig. 8 (Saravanan et al. 2016). The TiO_2_ nanoparticles were surface-treated with 3-Aminopropyltriethoxysilane as a coupling agent. Electrochemical resistance and static immersion tests were used to determine how resistant these surfaces were to corrosion and fouling. The embedding of the silane coupling agent effectively modified the surface characteristics of TiO_2_ nanoparticles, transforming them from hydrophilic to hydrophobic. This improved their dispersibility in epoxy nano-hybrid coatings and indicated appropriate interaction amongst the matrix and the inorganic materials. The uniform dispersion of APTES–TiO_2_ throughout the film enhances the coating’s hydrophobicity, repelling water and corrosion initiators, thus providing better corrosion protection attributes. After being submerged in a 3.5% NaCl solution for a month, the nano-hybrid coating APTES-TiO_2_ displayed a corrosion resistance of up to 108 Ω cm^2^. In addition, effective antifouling performance was shown during a static immersion test conducted in the ocean for up to 6 months.

TiO_2_ particle base coating was studied, and investigated its prospective applications in industrial heat exchangers (Wang and Liu 2011). In this research, liquid phase deposition was used to apply titanium dioxide thin films onto copper substrates, achieving film thicknesses in the nanometer range. The reduction in surface free energy exhibited a better non-fouling period on coated surfaces, which is more than that on untreated surfaces. Furthermore, the coating displayed exceptional anticorrosion resistance compared to uncoated material. Chuanbo Hu and colleagues developed a stearic acid-TiO_2_/zinc (STA-TZC) composite coating with self-cleaning properties, robustness, and corrosion resistance (Hu et al. 2021). The STA-TZC coating reflects superhydrophobic characteristics with a contact angle of 160°. The STA-TZC coating demonstrated better chemical and mechanical stability. Furthermore, the STA-TZC coating exhibited exceptional self-cleaning characteristics, resistance to corrosion (16349 Ω·cm^2^), and long-lasting superhydrophobic stability compared to pure zinc substrate (1716 Ω·cm^2^). The findings demonstrated that incorporating TiO_2_ nanoparticles into the porous zinc coating, followed by applying an STA layer, significantly enhanced the coating’s resistance to corrosion and fouling. Composite matrix made of CeO_2_-TiO_2_ metal oxides integrated into aluminium and examined its scope to resist corrosion and biofouling (Ashraf and Shibli 2008). According to the findings, CeO_2_ and TiO_2_ were determined to have the optimal concentrations of 0.2 and 0.1%, respectively. The findings presented here highlight the possible applications of CeO_2_ and TiO_2_-incorporated aluminium in maritime, highlighting the potential breadth of such applications. The TiO_2_ and CeO_2_ + TiO_2_- integrated showed a significant decrease in the quantity of microorganisms, indicating a strong biocidal property of the TiO with corrosion current density of 8.46 × 10^−7^ A cm^−2^ compared to bare pure aluminium sheets (1.13 × 10^−6^ A cm^−2^). In addition, the combination of hydroxyl radicals from metal oxides can also produce hydrogen peroxide, which is responsible for the biocidal effect.

Similar investigations were carried out by S.M.A. Shibli and team on the CeO_2_–TiO_2_ mixed oxide coating, and their efficacy towards antifouling and corrosion resistance was studied (Shibli and Chacko 2011). The inner layers of the coatings containing mixed oxide showed enhanced corrosion resistance. The neutral salt spray and EIS (electrochemical impedance spectroscopy) evaluations demonstrated the barrier protection attributes of the mixed oxide coatings. Besides corrosion resistance, the mixed metal oxide coating demonstrates excellent antifouling properties. In the case of zinc coatings, the surface was covered with a high concentration of colonies. However, when the CeO_2_-TiO_2_ oxide coating was exposed to microorganisms, no colonies were found on the surface after 144 h.

#### Nickel oxide (NiO)

Nickel’s corrosion resistance characteristics are particularly valuable in marine environments due to its ability to withstand exposure to seawater and other corrosive elements. This makes it a perfect candidate for a broad scope of utilisations in marine construction and industries, constituting desalination plants, pipework, and pumps. Formulating a passive film on the nickel surface further enhances its corrosion resistance, making it a preferred material for marine equipment and structures. Nickel’s resistance to corrosion also extends to its ability to resist fouling. This means that nickel is less likely to accumulate unwanted deposits or biological growth on its surface, which can further degrade the material and reduce its effectiveness. Zhifeng Huang and co-authors recently published a paper on developing stearic acid-modified porous nickel-based coating for magnesium alloy AZ31, aiming to achieve superhydrophobicity and improved corrosion resistance (Huang et al. 2023). The process involved electroless nickel-phosphorus plating, electrodeposition of porous nickel, and decoration with stearic acid to achieve good anti-corrosion properties. The composite exhibited a contact angle of 154.6° ± 3.7° and a water sliding angle value of 5.9° ± 1.7°, signifying an exceptionally high level of superhydrophobicity. Tafel fitting and EIS showed that the corrosion current density of the coating (*j*_*corr*_ = 1.41 × 10^−8^ A cm^−2^) was significantly minimal than that of the bare magnesium (*j*_*corr*_ = 7.4 × 10^−5^ A cm^−2^) alloy by three orders of magnitude. The contact angles in the composite were greater than 150°, showing excellent resistance to fouling.

Ni–P–nanoparticle composite coating has been studied for its potential to tackle corrosion and fouling resistance using online monitoring technology (Cao et al. 2023). The study establishes an online monitoring system to experimentally examine the corrosion resistance and heat transfer, fouling resistance of Ni–P coating and Ni–P–SiO_2_ coating in coal-fired boilers subjected to dew point corrosion and low-temperature ash deposition. The experimental tests, including electrochemical assessments and high-temperature experiments, indicated that the Ni–P-SiO_2_ composite coating demonstrates favourable effectiveness with regard to resistance against corrosion and fouling. The *R*_ct_ value for the Ni–P-SiO_2_ coating (50 g/L) is 1494 Ω cm^2^, significantly higher than ND steel by approximately 65 times. Raghupathy and team synthesised phase-segregated, nanocrystalline Ni-Ag coating through electrodeposition as a corrosion-resistant and biofouling coating (Raghupathy et al. 2016). The coating was subjected to sulphate-reducing bacteria (SRB), and the biofouling and corrosion resistance characteristics were much better compared to the copper substrate. Ni-Ag coatings demonstrate greater resistance to SRB biofilm formation compared to pure Cu substrate and Ni coating. The growth rate and coverage of biofilms decrease as the Ag content increases, providing further evidence of the antibacterial properties of Ag against sulphate-reducing bacteria. The intensity of biofilm formation directly impacts corrosion resistance after two months of exposure to sulphate-reducing bacteria. This research highlights the potential for developing Ni-Ag coatings as a viable option for addressing biocorrosion in marine settings.

Su Zhiwei and co-workers conducted similar research to investigate the influence of silver addition on nickel-based transition coatings in preventing biofouling and corrosion (Zhiwei et al. 2023). The count of sulphate-reducing bacteria attached to the coating surfaces decreased following exposure to the bacterial solution, and their outer layers disintegrated. Significantly, the coating retarded the growth of sulphate-reducing bacteria due to the shell-broken effect of silver. In addition, the current density of the composite declined by one order of 2.75 × 10^−6^ A cm^−2^ compared to steel plate 2.20 × 10^−5^ A cm^−2^. This indicates that the silver addition in the nickel-based transition coatings could enhance antimicrobial properties and effectively prevent biofouling and corrosion.

#### Silicon oxide (SiO_2_)

SiO_2_, with its cost-effectiveness, non-reactive nature, and water-attracting properties, is another suitable inorganic nanoparticle to minimise fouling and enhance the resistance to corrosion by modifying the surface morphology. The silica graphene oxide nanocomposite was synthesised, and its potential applications for resisting corrosion and fouling were successfully studied (Chen et al. 2023). The inclusion of Si-NP induces superhydrophobicity by presenting a roughness structure at two different scales, hence conferring long-lasting anti-corrosion characteristics. Adding GO/rGO (graphene oxide/reduced graphene oxide) significantly improves the anti-corrosion properties by creating a barrier layer within the coatings. This barrier layer slows down the diffusion of the corrosive substance, preventing it from contacting with the substrate. This sprayable anti-corrosion material can extend to different types of wire-mesh filters or metal alloy substrates, helping to avert fouling through its superhydrophobic properties and corrosion prevention. Similar investigations were carried out by Dawei Li and colleagues in developing SiO_2_ -GO-TiC nanocomposite coating for multifunctional applications (Li et al. 2022a). The coating that has been developed has exceptional super-hydrophobicity, with contact angles reaching up to 161.9°. The sliding angles are only 4.2°, and the adhesion value of microdroplets is around 16.8 ± 0.6 μN. The composite coating has exceptional persistence against corrosion and can endure exposure to corrosive substances for an extended period. The corrosion current density of the composite samples submerged for 21 days is marginally reduced to 2.14 × 10^−5^ A⋅cm^−2^ from 1.23 × 10^−9^ A⋅cm^−2^.

Xuezhun Gu and team fabricated vinyl-terminated polydimethylsiloxane, which was covalently embedded on the nano-SiO_2_ surface by a thiolene chemistry reaction, resulting in a highly effective anti-corrosion and fouling coating (Gu et al. 2023). The developed protective coating demonstrated consistent and outstanding efficacy in preventing fouling and corrosion, as confirmed by anti-corrosion and fouling tests conducted after submerging in a *Pseudoalteromonas* sp. culture medium for 14 days. The coating exceeded 98% bacterial attachment inhibition efficacy and an *R*_ct_ value of 1.57 × 10^9^ Ω·cm^2^ with 99.9% corrosion inhibition efficiency, making it highly effective in preventing bacterial adhesion and corrosion, contrary to uncoated steel sheets.

One of the challenges limiting the efficient use of geothermal energy is corrosion and fouling in heat exchangers and pipelines caused by geothermal water. Silica nanoparticle-based composites were investigated for their effectiveness in preventing fouling and corrosion in geothermal applications (Song et al. 2018). The SiO_2_ and SiO_2_-FPS (heptadecafluorodecyltri-isopropoxysilane) coating exhibited superior antifouling and corrosion properties when exposed to highly corrosive hot-dry-rock geothermal water with a total dissolved solids concentration of about 7000 mg/L. The electrochemistry investigations conducted on SiO_2_ and SiO_2_-FPS coatings in simulated geothermal water at a temperature of 423.15 K for 14 days indicate that both coatings can decline the corrosion rate by more than 60%. The application potential of SiO_2_ and SiO_2_-FPS coatings is promising for preventing corrosion and fouling in geothermal water with mild corrosiveness. Chen Ning and colleagues fabricated SiO_2_ coatings on copper and studied their suitability for corrosion and fouling resistance (Ning et al. 2012). A fraction of the SiO_2_-coated sample surface was encrusted with a fouling layer, whereas another section remained unchanged after the fouling trials. Hence, the SiO_2_ coatings possess the capability to inhibit the development of fouling. The SiO_2_-modified samples exhibited effective corrosion prevention immediately following immersion. However, after an extended period of submersion, their corrosion resistance was reduced due to the flaking of the SiO_2_ coating on the material’s layer.

### Multifunctional nanocoating based on nanoclay

The synthetic clay nanomaterial known as layered double hydroxide (LDH) has lately garnered interest from researchers for its wide range of applications. LDH is naturally present but not in large quantities. However, it is well acknowledged that LDH may be readily synthesised in laboratory environments. Multiple formulations of LDH can be synthesised economically. In a recent investigation carried out by Sepideh Pourhashem and the team successfully fabricated a superhydrophobic LDH-GO/DTMS (graphene oxide/silane) composite through an electrodeposition technique and examined its prospective against fouling and corrosion (He et al. 2022). The LDH-GO/DTMS coating provides remarkable fouling protection effectiveness of 99.98 ± 0.10% and significantly reduces the corrosion current density (4.87 × 10^−10^ A cm^−2^). by about four orders of magnitude in contrary to the uncoated aluminium alloy (2.09 × 10^−6^ A cm^−2^). LDH nanosheets can serve as a protective hurdle to avert corrosion. The inherent Cl^−^ containment property of LDH contributes to its ability to protect metals from corrosion. Incorporating graphene oxide into composite coatings extends the path for corrosive ions ascribable to its high aspect ratio and outstanding impermeability, thus enhancing anti-corrosion capabilities and long-term resistance through a labyrinth effect. Moreover, graphene oxide has natural anti-biofouling properties because of its unique synergistic effects. Introducing silane with an extended carbon chain structure enhances the hydrophobicity of the surface. The bovine serum albumin (BSA) adhesion capability on the coating is just 10% compared to that of bare aluminium surface. The LDH coatings are made hydrophobic by modifying the chain, which leads to strong water repellency and a physical barrier effect. This unique characteristic gives the coating excellent corrosion resistance and prevents BSA from adhering whilst combining LDH, GO, and DTMS, which produces composite coatings with excellent corrosion resistance and effective anti-biofouling attributes. The coating has excellent anti-biofouling properties, making it suitable for protecting aluminium alloys in maritime environments.

Yuxin Xiang and colleagues developed FeNi-LDH/ triethoxy(octyl)silane (TTOS) composite and studied its efficacy against multifunctional applications (Xiang et al. 2021). The composite reported superhydrophobic water contact angle reached 169 ± 2°, making it suitable for antifouling characteristics. Electrochemical investigations demonstrate that the corrosion inhibition rate of the coating attained 90.9% for 20 h of immersion with current density (*I*_*corr*_ = 1.02 × 10 A cm^−2^). Additionally, its impedance can be enhanced by two orders of magnitude compared to naked steel. The superhydrophobic surface enhances the corrosion and fouling resistance of the substrate. Zihao He et al. successfully designed Li-Al LDH films and explored their potential as a protective coating against fouling and corrosion (Li et al. 2021). The present study employed an easy in-situ growth technique to produce lithium-aluminium- LDH films. These films were later modified with 4-amino-2-((hydrazine methylene) amino)-4-oxobutanoic acid (also known as AOA acid) and 1H,1H,2H,2H-perfluorooctyltriethoxysilane, resulting in improved hydrophobicity, biofouling, and corrosion resistance. The coatings show better corrosion current density (*I*_*corr*_ = 0.010 μA cm^−2^) to corrosion compared to bare Al alloy (*I*_*corr*_ = 2.092 μA cm^−2^) in immersion tests, neutral salt testing, and EIS performed in 3.5 wt—% NaCl solutions. The intercalated AOA molecules provide further antimicrobial activity against SRB adhesion, *B. subtilis* and *E. coli*. The composite film exhibited better enhanced anticorrosion and fouling resistance than the uncoated alloy surface.

**Fig. 9:**
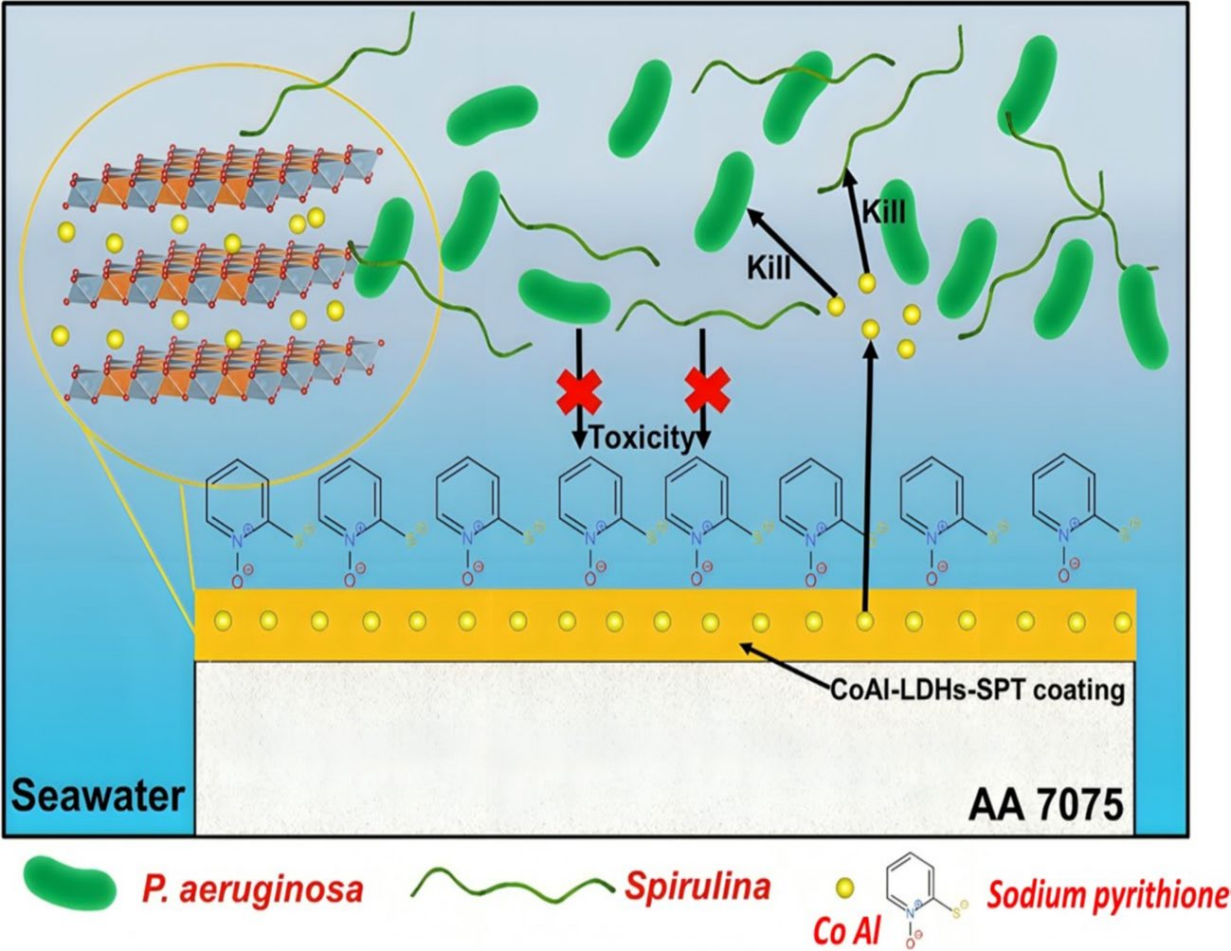
Co-Al LDHs-SPT mechanism against fouling and corrosion agents (Jian et al. 2022)

Jian and the team successfully fabricated Co-Al LDH followed by sodium pyrithione (SPT) modification to improve fouling and corrosion resistance (Jian et al. 2022). The charge transfer corrosion resistance of the Co-Al LDHs-SPT coating (*R*_ct_ = 7.8 × 10^6^ Ω·cm^2^) significantly improved in comparison to the bare AA 7075 (*R*_ct_ = 4.4 × 10^4^ Ω·cm^2^), with surface characterisation tests revealing minimal attachment of *Spirulina* and *Pseudomonas aeruginosa* cells to the CoAl-LDHs coating. Additionally, no biofouling cells were noticed for the CoAl-LDHs- sodium pyrithione coating. Sodium pyrithione functions as a corrosion inhibitor, effectively reducing the metal corrosion rate and eliminating microorganisms. The chemical bonding of SPT with Co on the surface acts as a corrosion inhibitor, as shown in Fig. 9. This attachment behaviour explains why the organic inhibitor can demonstrate potent corrosion inhibition, although it forms only a single layer on the metal surface. Pyrithione anions may be at threat of ion exchange with Cl^−^; however, this is hindered by the steric effect of adsorbed sodium pyrithione. As a result, the excellent anti-corrosion efficiency of Co-Al LDHs-SPT coating remains nearly unaltered during one month of artificial seawater testing. This indicates that the presence of SPT contributed to improved antibiofouling and anticorrosion characteristics in contrast to the CoAl-LDHs coating.

### Multifunctional nanocoating based on metal-organic framework

Metal–organic frameworks (MOFs) are a relatively new crystalline coordination material that combines organic linkers with metal ions or clusters. Marine biofouling and corrosion are two problems that MOFs show great promise in resolving (Han et al. 2022). Bioinspired liquid-infused surface (LIS) coating based on a MOF protective coating against biofouling and corrosion was investigated (Yu et al. 2022). The MOF-LIS compound effectively inhibits corrosion on the surface of copper. The typical measure of *R*_ct_ for MOF-LIS is 5.26 × 10^7^ Ω cm^2^, far surpassing the bare Cu value of 694.6 Ω cm^2^. This demonstrates a corrosion prevention effectiveness of up to 99.99%. When the composite coating was submerged in saltwater for eight days, the density of diatoms attached to the MOF-LIS material was much lower (7.33 × 10^4^ cells/cm^2^) compared to the bare copper surface (3.95 × 10^8^ cells/cm^2^). This demonstrates the strong ability of MOF-LIS to limit biofouling. Thus, the MOF-LIS is anticipated to be a very favourable option for preventing corrosion and biofouling in maritime environments. Ziyang Zhou and colleagues fabricated a graphene oxide hybrid with a ZIF-8 metal–organic framework for attaining long-term fouling and corrosion protection (Zhou et al. 2023d). The composite was later functionalised with silane moieties to improve the fouling and corrosion resistance characteristics. The antibacterial testing conducted on *Pseudomonas* sp. and the anti-fouling monitoring carried out in marine environments have noticed a reduced adhesion of bacteria and marine organisms on the composite material. In addition, the composite exhibited excellent corrosion resistance. Zhiqun Yu and his team developed biopolymer-based hydrogel and dense metal–organic framework coating for integrated antifouling and anti-microbial corrosion protection (Yu et al. 2023). The resulting coating, acting as a reservoir, displays a distinctive active defensive mechanism of intelligent breakdown of MOFs, leading to a pioneering performance in controlling broad-spectrum biofouling and corrosion. It is important to note that there is no observed adhesion of marine creatures or increased corrosion of metal substrates throughout the extended testing period in complex biological settings.

### Multifunctional nanocoating based on polymer composite

Nanocoatings based on polymer composites have gained significant attention as they offer unique properties and enhanced performance compared to conventional coatings. These nanocoatings, composed of a polymer matrix with dispersed nano-sized particles, exhibit improved corrosion resistance, mechanical strength, and antifouling. Investigations were carried out by Sara Fazli-Shokouhi and colleagues in polyaniline-modified graphene oxide (PANI-GON) nanocomposites in fouling and corrosion resistance (Fazli-Shokouhi et al. 2019). The protective coating exhibited a significantly high corrosion resistance, measuring 2.70 × 10^6^ Ω cm^2^ after being immersed in saline water for 192 h, as assessed by the EIS technique. The corrosion resistance was achieved by inhibiting diffusion processes against corrosive environments. Additionally, it displayed increased effectiveness in preventing fouling. The antifouling capability of the coatings was examined in a simulated environment. After ninety days of submersion in seawater with microorganisms, the epoxy/PANI-GON coating exhibited better antifouling performance than bare carbon steel. The corrosion protection is attained by filling the micropores of the epoxy matrix and altering the length and direction of corrosive species paths to the surface. The PANI-GON nanocomposites in the epoxy matrix restrict electrolyte diffusion, limiting electrolyte paths and increasing diffusion length. Additionally, constituting active coatings on the metal’s surface can achieve protection against Cl^−^ ions diffusion due to PANI-GON’s negative surface charge in the epoxy matrix. The potential of poly(aniline-co-nitroaniline) (CPANOA) nanoparticles was explored, and their effectiveness as a nanocoating for corrosion and fouling resistance was tested (Wang et al. 2020). The resistance of the composite coatings with PANI and CPANOA evaluated over 22 days, attributed to their promotion of a metal passivation layer formation that averts further corrosion by the corrosion medium, showcasing better corrosion current density and charge transfer resistance of 7.586 × 10^–14^ A/cm^2^ and 1.81 × 10^11^ Ω∙cm^2^ compared to 1.545 × 10^–12^ A/cm^2^ and 1.03 × 10^10^ Ω∙cm^2^ of pure epoxy coating. The sterilisation rate for a 2 mg/mL suspension of CPANOA nanoparticles is as high as 98.8% against *E. coli* and *B. subtilis,* which have broad-spectrum antibiotic resistance. The N^+^ structure in the composite coatings attracts bacteria to adhere to their surface and disrupts the cell membrane structure, creating excellent antifouling properties for the coating.

**Fig. 10:**
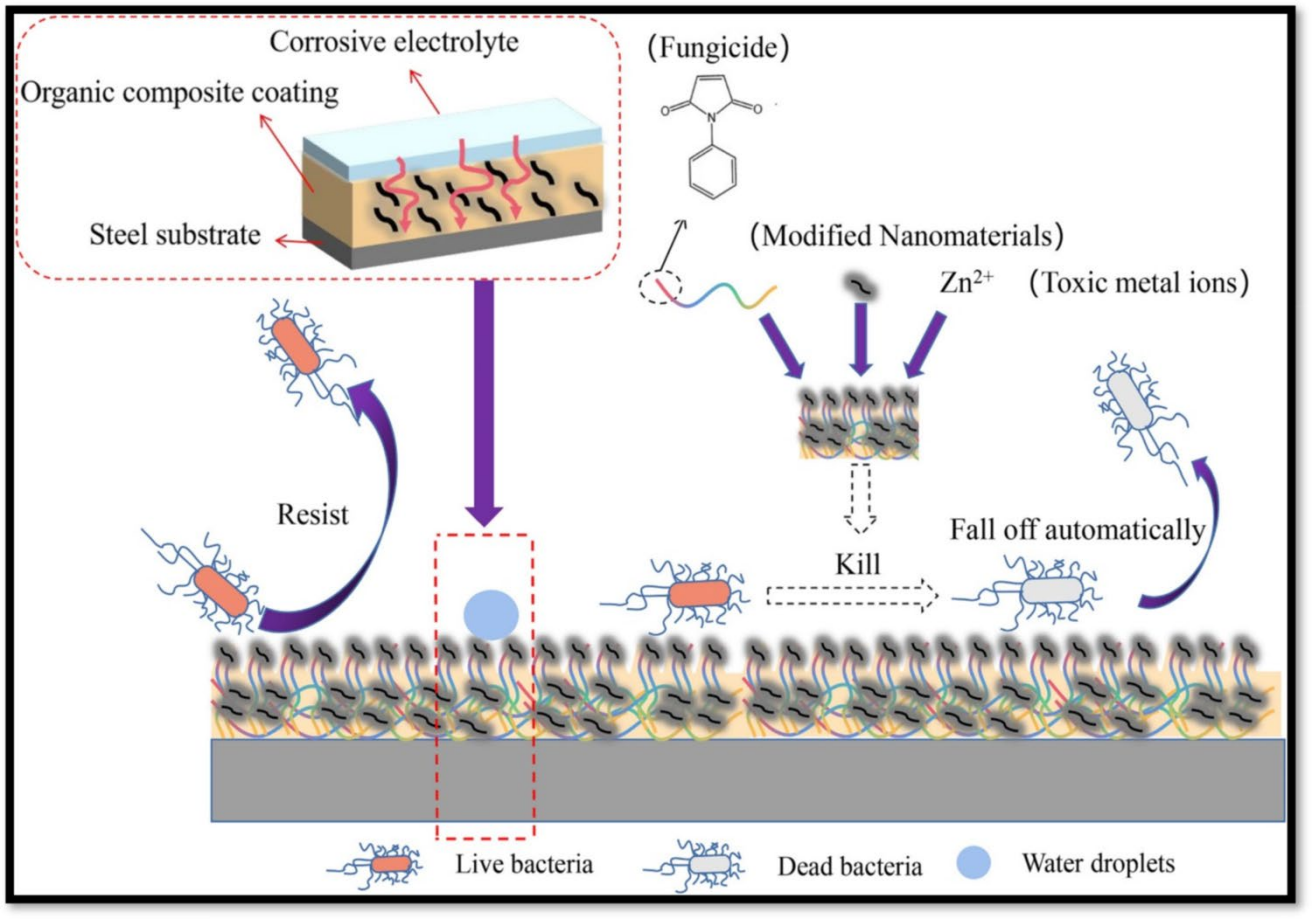
Anticorrosive and fouling mechanism of N-PMI modified PAZ nanocomposite coatings (Zhou et al. 2023a)

Acrylic resin modified with zinc ions and a structural functional monomer (N-phenyl maleimide; N-PMI) was efficiently created through free radical solution polymerisation (Zhou et al. 2023a). The composite coating samples were prepared by dispersing fluorinated silica-coated hydroxylated multi-walled carbon nanotubes nanocomposite (F-MWCNTs-OH@SiO_2_) in PAZ/N-PMI resin. The composite coating possesses antibacterial, anti-corrosive, and self-repairing capabilities, as shown in Fig. 10. As a result, it has significant potential for use in maritime equipment and the shipyard industry, offering valuable insights into marine antifouling and anti-corrosion technology. After being submersed for ten days, the composite coating retained a *Z*_*f*_ = 0.01 Hz (2.453 × 10^9^ Ω∙cm^2^), showcasing minimal diatom attachment and demonstrating strong resistance to algae attachment. Additionally, it exhibited notable antibacterial effects against *E. coli* and *S. aureus*. Shatakshi Verma and colleagues developed multifunctional polydimethylsiloxane (PDMS)-epoxy-zinc oxide nanocomposite coatings for maritime applications (Verma et al. 2019). The enhancement in water resistance was accompanied by the improved ability to prevent corrosion, showing 98.8% inhibition efficiency compared to the control coating and a minimal corrosion rate of 0.12 × 10^−3^ mm/year compared to uncoated mild steel (1.09 mm/year). The Taber abrasion resistance and pull-off adhesion strength outcomes noticed an increase of 34.7% and 150.7%, individually. The study demonstrated that the nanocomposite coating efficaciously prevented the attachment of biofouling organisms and marine bacteria for over eight months. This eco-friendly and effectual nanocomposite shows potential as a better coating compared to uncoated mild steel samples for preventing corrosion and fouling in marine applications. Bilayer coating based on polybenzoxazine/zeolite nanocoating composite was investigated for corrosion and fouling resistance (Wang et al. 2022a). In this research, a dual-layer superhydrophobic anti-corrosion coating with varying concentration gradients was developed. Benzoxazine was utilised as the matrix, whilst waste fly ash-prepared zeolite served as the inorganic filler. The composite coating demonstrated a superhydrophobic surface with a WCA measuring 158.8° ± 0.9°. The impedance value of the developed coating is 2.677 × 10^8^ Ω, which is four magnitudes higher than that of the benzoxazine coating. The combination of benzoxazine and zeolite coatings showed improved resistance to fouling and corrosion, leading to increased utilisation of fly ash and the development of a new form of anti-corrosion coating.

Prospectives of block copolymer (PMBTMA-co-PSPMA)-functionalised Ti_3_C_2_Tx as protective nanocoating for fouling and corrosion resistance were investigated (Wang et al. 2022b). The Ti_3_C_2_Tx surface was initially modified with the anticorrosion agent poly(2-mercaptobenzothiazole), followed by grafting the antifouling agent poly(3-sulfopropyl methacrylate potassium) employing surface-initiated atom transfer radical polymerisation. This process resulted in obtaining the block copolymer-functionalised Ti_3_C_2_Tx, abbreviated as TMS. The impressive antibacterial attributes of TMS and the adequate surface hydration of the anionic polymer brushes PSPMA give the synthesised TMS-based nanocomposite coating (TMS/EP) strong antifouling performance, with over 55% bacteria removal and a microalgae removal rate of up to 71%. Furthermore, TMS/EP exhibits remarkable resistance to corrosion due to the combined hurdle impact of Ti_3_C_2_Tx nanosheets and the gradual release of the corrosion inhibitor MBT throughout the corrosion cycle. In addition, the TMS nanohybrid coating demonstrated outstanding corrosion resistance, exhibiting an impedance modulus of 3.73 × 10^7^ Ωcm^2^ after being immersed for fifteen days. This is attributed to the practical synergistic anticorrosion barrier effect of Ti_3_C_2_Tx nanosheets.

**Fig. 11:**
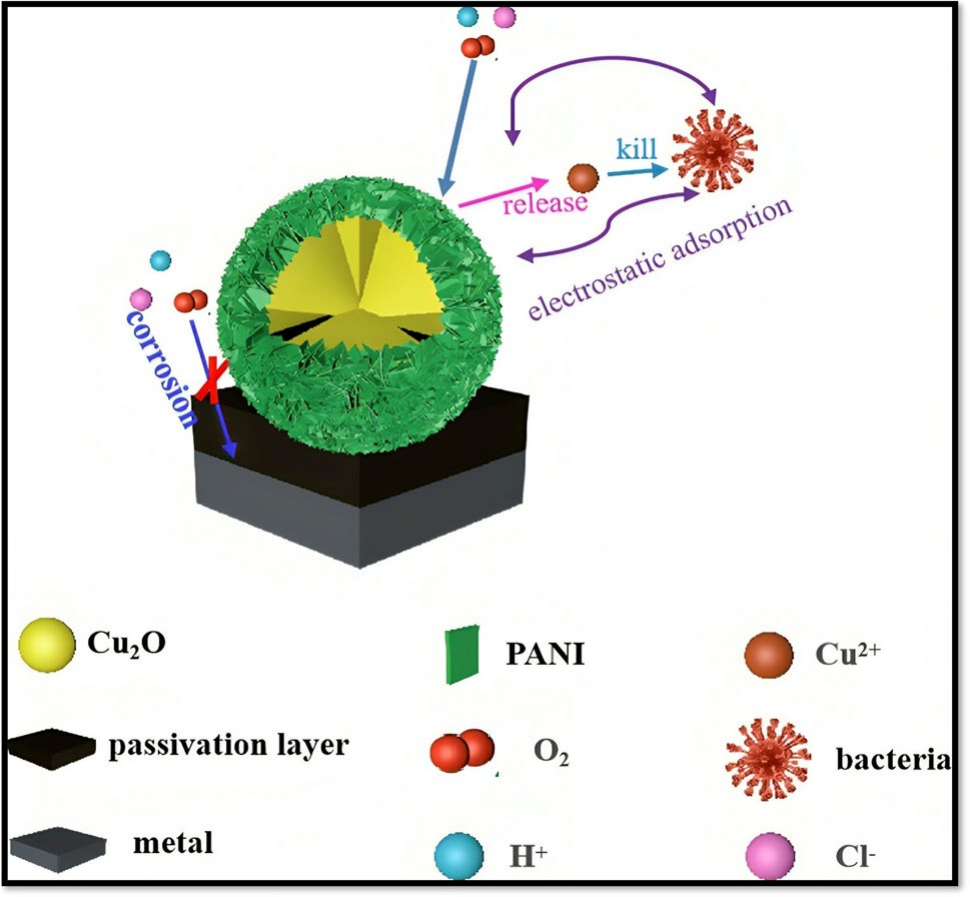
Schematic illustration of cuprous oxide/polyaniline composite as a protective coating against corrosion and fouling (Zhao et al. 2022)

Yunyan Zhao and colleagues developed cuprous oxide/polyaniline composites and studied their prospective application against fouling and corrosion (Zhao et al. 2022). Cu_2_O/PANI-2 coating demonstrated outstanding corrosion resistance, showing a corrosion current density of 6.49 × 10^−5^ μA/cm^2^ and a low corrosion rate of 7.55 × 10^–4^ μm/year compared to uncoated sample (50 μm/year and current density of 4.30 μA/cm^2^). The structural layout maximises the electrostatic attraction of PANI shell to Gram-negative *E. coli* to reduce the pathways for Cu^2+^ function. The effective use and gradual release of antifouling agents can enhance bactericidal effectiveness, as shown in Fig. 11. Consequently, the bacteriostasis rate of Cu_2_O/PANI-2 coating (91.9%) is much better than that of conventional Cu_2_O coating. Similarly, Hongli Tian and colleagues studied bioinspired Coating with Doped Polyaniline and TO@CA (doped polyaniline and nano-titanium dioxide) self-healing nanocapsules (Tian et al. 2023). The nanocapsule coating antifouling performance was evaluated for a longer duration, and it was noticed that fewer algae were attached to the surface. In addition, the nanocomposite exhibited excellent resistivity against corrosion with a charge transfer resistance *R*_ct_ of 1.034 × 10^7^ Ω·cm^2^ compared to epoxy resin coating of 1.517 × 10^–5^.

## Performance evaluation in field applications

Studying numerous articles related to integrated multifunctional nano coating against fouling and corrosion. It is noted that the durability of these nano coatings is a crucial factor in their effectiveness and long-term performance. This is especially important in marine, oil, gas, and automotive industries, where materials are exposed to harsh environmental conditions and corrosive substances. However, the majority of the works lack real-time environmental field studies. During the antifouling studies in laboratory conditions, the nanocoatings were exposed to certain algae and bacteria specimens. Although initial laboratory studies provide valuable insights and initial assessments of the durability of nanocoatings, it is essential to conduct real-time environmental field studies to validate their durability in practical applications. The marine ecosystem is complicated, with numerous organic and inorganic substances and various fouling organisms, making it crucial to assess the durability of nanocoatings in real-world scenarios.

For example, research carried out by Liu et al., where the authors investigated the durability of zwitterionic polymer coatings for fouling resistance (Liu et al. 2019). During the laboratory investigation, the coating showed promising resistance against red algae Porphyridium sp. On the marine field test, the composite was covered with mud and adhesion of the *Callysponginvaginalis larva*. The researchers acknowledged that real-world conditions could differ significantly from the controlled laboratory setting. Therefore, further research and testing are needed to assess the durability of these nano coatings in real-world situations and to ensure their effectiveness over an extended period. In conclusion, whilst laboratory studies provide important insights into the durability of nanocoatings, real-time environmental field studies are necessary to fully evaluate their long-term performance and effectiveness in practical applications, especially in industries with harsh environmental conditions.

The integration of nanocoating towards multifunctional protection against fouling and corrosion is still an emerging research field. Notably, there is limited literature on nanocoating for integrated protection against corrosion and fouling. There has been growing attention to developing nanocoatings that provide both antifouling and anticorrosion properties since 2016 and are anticipated to extend in the future. As discussed earlier, the majority of the studies focus on laboratory, aquarium, and river environments. However, conducting field studies in diverse backgrounds, such as oceanic environment ecosystems, is essential. This will enable a comprehensive understanding of the durability and performance of integrated nanocoatings as they are exposed to real-world conditions and multiple types of fouling and marine organisms.

Even though there is no universal or standard protocol regarding the duration of marine field tests. Several variables must be taken into account during on-site investigations. The antifouling and corrosion samples must be positioned at a depth ranging from 2 to 5 m. Additionally, all sample substrates should be arranged in a sequential manner at the same depth. Furthermore, it is optimal to do sample testing in three or more sites to comprehend the impact of spatiotemporal differences. Examining the effect of changes in environmental variables, such as temperature, pH, and salinity, on nanocoatings may provide significant information on their performance in various scenarios and aid in assessing their appropriateness for prolonged usage in real-world applications. The substrate samples must undergo analysis at four-week intervals to evaluate the performance of the material coating over time. Additionally, the analysis should be extended to durations of six months or longer to assess the long-term durability and effectiveness of the nanocoatings accurately. During the sample maintenance, it is essential to clean it exclusively using a fine saltwater spray to ensure that no biofouling or other live creatures are adhered to the surface and eliminated. Furthermore, it is crucial to observe and classify bioorganisms that are attached to the surface, including slime, diatoms, algae, invertebrates, and macroalgae.

## Challenges

As discussed in the previous sessions, the main challenge associated with coating are limited to its field investigations. Further research and development efforts are necessary to address this limitation and provide more comprehensive insights into nanocoating performance and practical applications as a protective coating against corrosion and fouling. Numerous reports on the mismatching of nanocoating efficacy in laboratory settings versus real environmental conditions highlight the need for further study and validation. Moreover, the durability and stability performance, in particular the long-term stability efficacy of the nanocoatings, are poorly studied and require a comprehensive understanding. Furthermore, the cost-effectiveness and scalability of producing nanocoatings on a commercial scale should be thoroughly evaluated.

Most of the research investigations highlighted the hydrophobic nature of the coating as the main characteristic that prevents the adhesion of fouling and other microorganisms. However, the adhesion behaviour depends on the fouling specimens and microorganisms as well as the specific environmental conditions. Some reports state that the hydrophilic nature of the nanocoating surface can enhance fouling resistance by disrupting the biofilm formation, which mismatches the general research conclusion that only the hydrophobic nature prevents fouling (Yin et al. 2021). These claims need to be clarified and confirmed with fouling agent adhesion tests, and their performance in marine environments needs to be evaluated. In addition, the low bonding strength of some nanocoatings to the substrate surface is an additional challenge that must be addressed for long-term durability and effectiveness. The minimal surface energy on the coating surface may also lead to issues such as poor adhesion and reduced performance in specific applications.

Slippery liquid-infused porous surfaces based nano-composites have certain limitations in their practical suitability due to their poor mechanical stability and strength. In addition, the complexity and expensive process of the fabrication of these surfaces hinders their widespread applications. Lastly, it is critical to evaluate and investigate the potential environmental impacts of nanocoatings. These issues include the release of nanoparticles into the ecosystem, the persistence and accumulation of nanocoating materials in ecosystems, and potential harm to marine life. Graphene and other carbon-based nanomaterials may seriously threaten nontargeted organisms and ecosystems, and need to evaluate their toxicity release.

In conclusion, whilst nanocoatings promise improved durability and performance, several challenges must be addressed before widespread implementation. These challenges include validating efficacy in real environmental conditions, investigating durability and stability performance, evaluating cost-effectiveness and scalability, understanding the adhesion behaviour of the coating, addressing issues with substrate bonding strength, and assessing potential environmental impacts. In addition, there is a need to discover and identify efficient, low-cost, and simple synthesis techniques for nanocoatings that can overcome the limitations of current fabrication processes. Overall, further research and innovations are needed to overcome these challenges and ensure that nanocoatings can be effectively utilised in a broad range of applications with minimal environmental impact.

## Concluding remarks and perspectives

This review paper presents the performance and challenges of developing integrated multifunctional antifouling and anticorrosion nanomaterial-based coatings. This review highlights the functionality of integrating nanomaterials such as carbon nanomaterials, graphene oxide, nanometal oxides, nanoclays, and polymers in coating formulations for integrated multifunctional nanocoating applications. Integrated multifunctional nanocoating has immense prospective over traditional antifouling or anticorrosion coating. Owing to the intricate nature of maritime environment, integrated multifunctional nanocoating must have extensive stablity and enduring durability.

It is also necessary to consider these nanomaterials environmental impact and safety to ensure sustainable coating solutions. Moreover, future research should address on optimising the performance and durability of these integrated coatings and exploring new nanomaterials and synthesis approaches. Currently, the development of integrated antifouling and anticorrosion nanocoatings is still in the premature stage. The primary design concept of the integrated multifunctional coating is the coalescing of antifouling and anticorrosion characteristics into a single coating. Thus, there is a necessity for further research in the development of novel combinations; more efficient and effective integrated antifouling and anticorrosion nanomaterial-based coatings are anticipated to emerge in the time to come. In addition, it is very critical and fundamental to understand the long-term performance and environmental impacts of these integrated nanocoatings in real-world applications. In conclusion, the advancement of integrated antifouling and anticorrosion nanomaterial-based coatings is promising and will advance with the developments of material science.
